# Biological vs Synthetic Mesh in Laparoendoscopic Inguinal Hernia Repair: The BIOLAP Randomized Clinical Trial

**DOI:** 10.1001/jamasurg.2025.4071

**Published:** 2025-10-08

**Authors:** Claudia Simone Seefeldt, Judith Knievel, Muneer Deeb, Niels-Torsten Hoedt, Claudia Rudroff, Ralf Essen, Dirk Antoine, Gernot Maximilian Kaiser, Christoph Andreas Jacobi, Wilhelm Gross-Weege, Wolfgang Gänsler, Bernhard Johannes Lammers, Christoph Bonk, Frauke Hildebrandt, Thomas Carus, Moritz Meyer, Mathias Bebobru, Alexander Krökel, Jan Dornbusch, Mark Schneider, Nicola Cerasani, Bernd Stechemesser, Rolf Lefering, Dirk Rolf Bulian, Michael Alfred Ströhlein, Anna Rieger, Jonas Lange, Jürgen Stephan Meyer-Zillekens, Claus Ferdinand Eisenberger, Markus Maria Heiss

**Affiliations:** 1 Department of Abdominal, Tumor, Transplant and Vascular Surgery, Cologne Merheim Medical Center, Kliniken der Stadt Köln, Witten/Herdecke University, Cologne, Germany; 2 Department of General and Visceral Surgery, Ammerland Klinik, Westerstede, Germany; 3 Department of General and Visceral Surgery, Evangelisches Diakonissenkrankenhaus, Leipzig, Germany; 4 Department of Visceral Surgery and Functional Surgery of the Lower Gastrointestinal Tract, Evangelisches Klinikum Köln Weyertal, Cologne, Germany; 5 Department of General and Visceral Surgery, GFO Kliniken Rhein Berg, Bergisch Gladbach, Germany; 6 Department of General, Visceral and Thoracic Surgery, Klinikum Leverkusen, Leverkusen, Germany; 7 Department of General and Visceral Surgery, St Bernhard-Hospital Kamp-Lintfort, Kamp-Lintfort, Germany; 8 Department of Surgery, Dreifaltigkeits-Krankenhaus, Wesseling, Germany; 9 Department of General and Visceral Surgery, St Elisabeth-Krankenhaus, Dorsten, Germany; 10 Department of General, Visceral and Vascular Surgery, Josephs-Hospital Warendorf, Warendorf, Germany; 11 Department of General and Visceral Surgery, Lukaskrankenhaus Neuss, Neuss, Germany; 12 Department of General and Visceral Surgery, St Barbara-Klinik Hamm-Heessen, Hamm, Germany; 13 Department of General and Visceral Surgery, GRN Klinik Weinheim, Weinheim, Germany; 14 Department of General and Visceral Surgery, Elisabeth-Krankenhaus Thuine, Thuine, Germany; 15 Department of General and Visceral Surgery, St Marien-Krankenhaus Ahaus, Ahaus, Germany; 16 Department of General and Visceral Surgery, Helios Klinik Attendorn, Attendorn, Germany; 17 Department of General and Visceral Surgery, Eduardus-Krankenhaus Köln-Deutz, Cologne, Germany; 18 Department of General, Visceral and Vascular Surgery, KMG Klinikum Luckenwalde, Luckenwalde, Germany; 19 Department of General, Visceral and Transplantation Surgery, University Hospital RWTH Aachen, Aachen, Germany; 20 Department of General and Visceral Surgery, Johanniter-Krankenhaus Bonn, Bonn, Germany; 21 Hernia Center Cologne, PAN-Klinik, Cologne, Germany; 22 Institute for Research in Operative Medicine, Witten/Herdecke University, Cologne, Germany

## Abstract

**Importance:**

Chronic postoperative pain and recurrence are relevant complications after inguinal hernia repair. Biological meshes have been proposed as a resorbable alternative to synthetic implants that may reduce postoperative pain.

**Objective:**

To compare postoperative pain and recurrence rates between biological and synthetic meshes in laparoendoscopic inguinal hernia repair using a self-controlled design.

**Design, Setting, and Participants:**

The Biological vs Synthetic Mesh in Laparoendoscopic Inguinal Hernia Repair (BIOLAP) trial was a multicenter, randomized, self-controlled clinical trial conducted between August 2017 and February 2021 across 21 certified German hernia centers. A total of 491 adult patients with primary bilateral inguinal hernias underwent laparoendoscopic repair using 1 biological mesh and 1 synthetic mesh. Data analysis was performed from July 2023 to June 2024.

**Intervention:**

Patients were randomized to receive a biological or synthetic mesh on the right side; the contralateral side received the alternate material. All patients underwent standardized laparoendoscopic repair (transabdominal preperitoneal plasty or total extraperitoneal plasty), and both patients and assessors were blinded to mesh assignment.

**Main Outcomes and Measures:**

The coprimary outcomes were pain intensity at 6 months (measured using the visual analog scale) and hernia recurrence at 2 years. Secondary outcomes included seroma, hematoma, surgical site infection, and patient satisfaction.

**Results:**

Among 491 patients (mean [SD] age, 58.5 [14.1] years; 457 [93.1%] male), there was no significant difference in pain at 6 months between biological and synthetic meshes (mean [SD] visual analog scale score at rest, 0.3 [0.9] for both; *P* = .76). However, the 2-year recurrence rate was significantly higher for biological meshes (53 recurrences [11.2%]) compared with synthetic meshes (12 recurrences [2.5%]) (*P* < .001). Seroma rates were also significantly higher with biological meshes than with synthetic meshes (164 patients [33.4%] vs 106 patients [21.6%], respectively; *P* < .001).

**Conclusions and Relevance:**

Biological meshes did not reduce postoperative pain but were associated with significantly higher recurrence and seroma rates compared with synthetic meshes. These findings do not support the routine use of biological meshes in laparoendoscopic inguinal hernia repair.

**Trial Registration:**

German Clinical Trials Register Identifier: DRKS00010178

## Key Points

**Question:**

Does the use of resorbable biological meshes reduce postoperative pain without increasing recurrence rates in laparoendoscopic inguinal hernia repair?

**Findings:**

In this multicenter, randomized, self-controlled clinical trial of 491 patients with bilateral inguinal hernias, biological mesh implants did not reduce postoperative pain compared with synthetic meshes. However, biological meshes were associated with a significantly higher recurrence rate (11.2% vs 2.5%) and increased seroma formation.

**Meaning:**

The use of biological mesh in laparoendoscopic inguinal hernia repair did not confer a benefit in pain reduction and resulted in inferior outcomes regarding hernia recurrence; therefore, synthetic mesh should remain the standard of care.

## Teaser

This randomized clinical trial compares postoperative pain and recurrence rates between biological and synthetic meshes in laparoendoscopic inguinal hernia repair.

## Introduction

Inguinal hernia repair is one of the most common operations globally.^1,2^ Up to 30% of patients who present for surgical treatment have bilateral inguinal hernias.^3,4^ Laparoendoscopic mesh implanting procedures result in less postoperative pain and wound infections compared with open surgical techniques and are recommended especially for bilateral repairs in adults.^5^ Transabdominal preperitoneal plasty (TAPP) and total extraperitoneal plasty (TEP) with synthetic mesh implantation are the established standard procedures for laparoendoscopic inguinal hernia repair and equivalent in terms of recurrence rate and postoperative pain.^6^ However, a recurrence rate of 1% to 10% is a persisting clinical problem,^7^ as is chronic inguinal pain with a frequency of 11% to 54%.^8^ Both complications are influenced by the implant used.^9,10,11^

Biological meshes are collagenous xenogeneic matrices that were developed as a resorbable implant alternative and are supposed to induce stabilizing tissue remodeling via cell invasion and collagen deposition without a lifelong remaining foreign body.^11^ Until the start of the BIOLAP trial, there were no reasonably sized randomized clinical trials that directly compared biological vs synthetic meshes.^12,13^

In bilateral-occurring diseases, a self-controlled study design is possible in which each participant receives 2 alternative treatments simultaneously and thereby functions as an intrinsic control.^14,15^ As most confounding factors thus influence both study arms equally, an ideal comparison of outcomes, including subjective factors like pain or patient satisfaction, between 2 therapeutic options is possible.

The Biological vs Synthetic Mesh in Laparoendoscopic Inguinal Hernia Repair (BIOLAP) randomized clinical trial aimed to compare biological vs synthetic mesh implants in laparoendoscopic inguinal hernia repair. We hypothesized that resorbable biological meshes reduce postoperative pain (superiority) without increasing the recurrence rate (noninferiority).

## Methods

### Study Design

This randomized, self-controlled clinical trial was conducted in 21 German surgery departments with a special focus on hernia surgery, which guarantees a high number of patients and ensures surgical quality. Ethical approval from the lead Ethical Committee of Witten/Herdecke University was granted on July 4, 2017, with additional approval from participating centers. The trial protocol (available in Supplement 1) was published in January 2019.^16^ The statistical analysis plan is available in Supplement 2. A list of the study investigators and sites appears in the eAppendix in Supplement 3. The study followed the Consolidated Standards of Reporting Trials (CONSORT) reporting guidelines for randomized clinical trials.^17^ Data analysis was performed from July 2023 to June 2024.

### Participants

Adult patients with primary bilateral inguinal hernias were screened by local investigators. All study participants provided written informed consent before randomization. Exclusion criteria were essentially limited to recurrent hernias, incarcerated hernias, and acute systemic infection.^16^

### Randomization

Each patient received a biological implant on one side and a synthetic mesh on the other. Randomization of the right side (biologic or synthetic mesh) was performed up to 72 hours before surgery. Each patient received the other mesh on the left side. Randomization was stratified by center and performed as block randomization with varying block sizes. Both patients and clinicians were blinded regarding the location of each mesh type, except for the operating surgeon. Unblinding was only permitted in case of recurrence.

### Procedures

Enrolled patients underwent routine laparoendoscopic bilateral hernia surgery according to the guidelines of the International Endohernia Society, except for the implant materials. Both mesh materials were implanted simultaneously using the same technique (TEP or TAPP). As the study was conducted independently of mesh manufacturers, participating centers were permitted to use their standard meshes within the given criteria.^16^ These were defined as a perforated, non–cross-linked, acellular, collagenous matrix for the biological mesh and large-pored, lightweight synthetic meshes made of polypropylene, polyester, or polyvinylidene fluoride.

During each follow-up visit (discharge and 1 week, 6 months, 1 year, and 2 years postoperatively), pain, recurrence, complications, and patient satisfaction were assessed separately for each side. Pain was measured with the visual analog scale (VAS; scores ranged from 0-10), and recurrence was confirmed through clinical examination and imaging (ultrasonography, computed tomography, or magnetic resonance imaging). Follow-up visits were performed by physicians of the study team other than the operating surgeon. All data were collected prospectively by study team members of the participating centers and confirmed by independent on-site monitor visits.

### Outcomes

Local pain after 6 months and recurrence within 2 years were defined as primary end points. Complications and patient satisfaction were recorded as secondary end points at every follow-up visit.

### Statistical Analysis

Since 2 primary end points were considered, the *P* value was adjusted according to Bonferroni (*P* < .025 for statistical significance). Pain intensity at 6 months was expected to differ by at least 0.5 point (0-10 scale; SD, 1.0 point; Wilcoxon signed rank test; 2-sided α = .025; power, 90%). The recurrence rate was tested for noninferiority with a maximum tolerable difference of +3%. The sample size was calculated separately for each primary end point (131 patients for pain and 451 for recurrence), and the larger sample size was used for the study. Assuming a 10% loss to follow-up, a total of 496 randomized patients were required.^16^ SPSS version 29.0 statistical software (IBM) was used.

The study population comprised all randomized participants who received both implant types. All end points were analyzed according to intention to treat (ITT). The unit of analysis was the hernia, not the patient. In cases of unilateral recurrence, the contralateral side remained for further follow-up. In the absence of follow-up data, or a prior recurrence, pain data were imputed according to the last-observation-carried-forward method, but only up to 6 months postoperatively. The evaluation of recurrence required a minimum observation period of 6 months. The difference in recurrence rates was expressed as the 95% CI.

## Results

**Figure 1.**
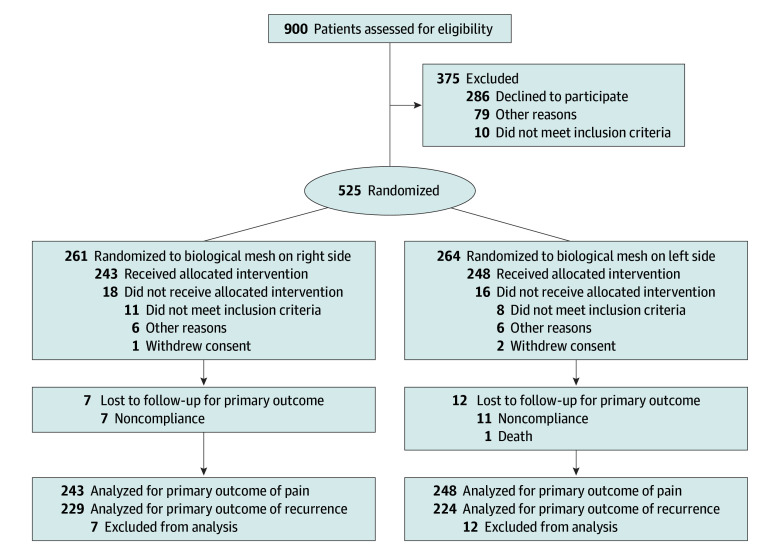
CONSORT Flow Diagram of the Biological vs Synthetic Mesh in Laparoendoscopic Inguinal Hernia Repair (BIOLAP) Randomized Clinical Trial

In this study, 525 patients were enrolled between August 2017 and February 2021, while 34 patients were excluded from analysis after randomization because surgery was not performed (Figure 1). In 5 cases, the surgeon implanted the mesh material contrary to randomization, reversing the left and right side. Since these patients still received both materials, they remained in the ITT population, with the randomized side assignments maintained.

### Patient and Hernia Characteristics

A total of 491 patients aged between 19 and 91 years were included (mean [SD] age, 58.5 [14.1] years), with 457 (93.1%) being male. The physical status ranged from an American Society of Anesthesiologists classification of I to III. The most common preexisting conditions were diabetes (n = 35), history of myocardial infarction (n = 15), chronic pain (n = 11), tumor diseases (n = 7), and kidney insufficiency (n = 9). A total of 149 patients (30.3%) had prior abdominal surgery, including appendectomy, cholecystectomy, or prostatectomy (Table 1). Due to the self-controlled study design, each patient contributed data to both study arms. All subtypes of the European Hernia Society’s primary inguinal hernia classification^18^ were included in the trial (eTable 1 in Supplement 3). Of the 982 hernias, 504 (51.3%) were solely of indirect anatomy, with L2 being the most common configuration (275 hernias [28.0%]). A total of 234 (23.8%) were combined hernias.

**Table 1.** Patient Characteristics, Operative Details, and Complications

Characteristic	Patients, No. (%) (n = 491)
Age, y	
Mean (SD)	58.5 (14.1)
Median (IQR) [range]	58.6 (50.5-68.6) [19-91]
Sex	
Female	34 (6.9)
Male	457 (93.1)
BMI, mean (SD)	25.8 (3.4)^a^
ASA classification	
I	161 (32.8)
II	272 (55.4)
III	58 (11.8)
Previous abdominal surgery	
Appendectomy	89 (18.1)
Cholecystectomy	27 (5.5)
Prostatectomy	5 (1.0)
Spinal surgery	13 (2.6)
Other	29 (5.9)
Operative time, median (IQR), min	75 (62-93)
Operative technique	
TAPP	339 (69.0)
TEP	152 (31.0)
Length of hospital stay, median (IQR), d	2.0 (1-2)
Simultaneous procedure	95 (19.3)
Umbilical hernia repair	80 (16.3)
Repair of other hernias	4 (0.8)
Vasectomy	4 (0.8)
Appendectomy	1 (0.2)
Other	7 (1.4)
Intraoperative complication	21 (4.3)
Bleeding	14 (2.9)
Mild	13 (2.7)
Moderate	1 (0.2)
Peritoneal tear	4 (0.8)
Visceral injury	2 (0.4)
Bladder	1 (0.2)
Bowel	1 (0.2)
Difficult mesh fixation	2 (0.4)
Postoperative complication prior to discharge	55 (11.2)
Hematoma	23 (4.7)
Seroma	17 (3.5)
Systemic infection	1 (0.2)
Bowel paralysis	1 (0.2)
Other	18 (3.7)

Abbreviations: ASA, American Society of Anesthesiologists; BMI, body mass index (calculated as weight in kilograms divided by height in meters squared); TAPP, transabdominal preperitoneal plasty; TEP, total extraperitoneal plasty.

^a^ Data missing from 1 patient (n = 490).

### Perioperative Data

Among the 491 patients overall, 339 patients (69.0%) received the TAPP technique and 152 patients (31.0%) received TEP repair. In 95 cases (19.3%), simultaneous procedures, such as umbilical hernia repair, repair of other hernias, or vasectomy, were performed. Intraoperative adhesiolysis was necessary for 115 patients (23.4%), 2 of whom also required prophylactic serosa suturing. Intraoperative complications were reported for 23 patients (4.7%), most commonly mild bleeding. The median (IQR) operation time was 75 (62-93) minutes, including simultaneous procedures. The longest operation lasted 261 minutes, and the surgeon reported problems with biological mesh fixation. The shortest operation time for bilateral hernia repair was 32 minutes (Table 1).

The median (IQR) time to discharge was 2 (1-2) days. Five patients underwent outpatient surgery, while 8 patients were discharged after more than 6 days. All hospital stays longer than 2 days were associated with 1 or more complications. Complications prior to discharge were reported for 55 patients (11.2%), most commonly hematomas and seromas of the groin (Table 1; eTable 2 in Supplement 3). Five patients required revision surgery: 2 for exploratory laparoscopy without further intervention and 3 for hematoma evacuation.

### Mesh Material and Fixation

Three different biological implants and 9 different synthetic meshes were used across the participating centers (eTable 3 in Supplement 3). Mesh fixation was conducted in 47.5% of hernia repairs, in most cases with fibrin glue (87.8%).

### Primary Outcomes

This study examined 2 primary end points in parallel: pain intensity at 6 months and recurrence rate at 2 years.

**Figure 2.**
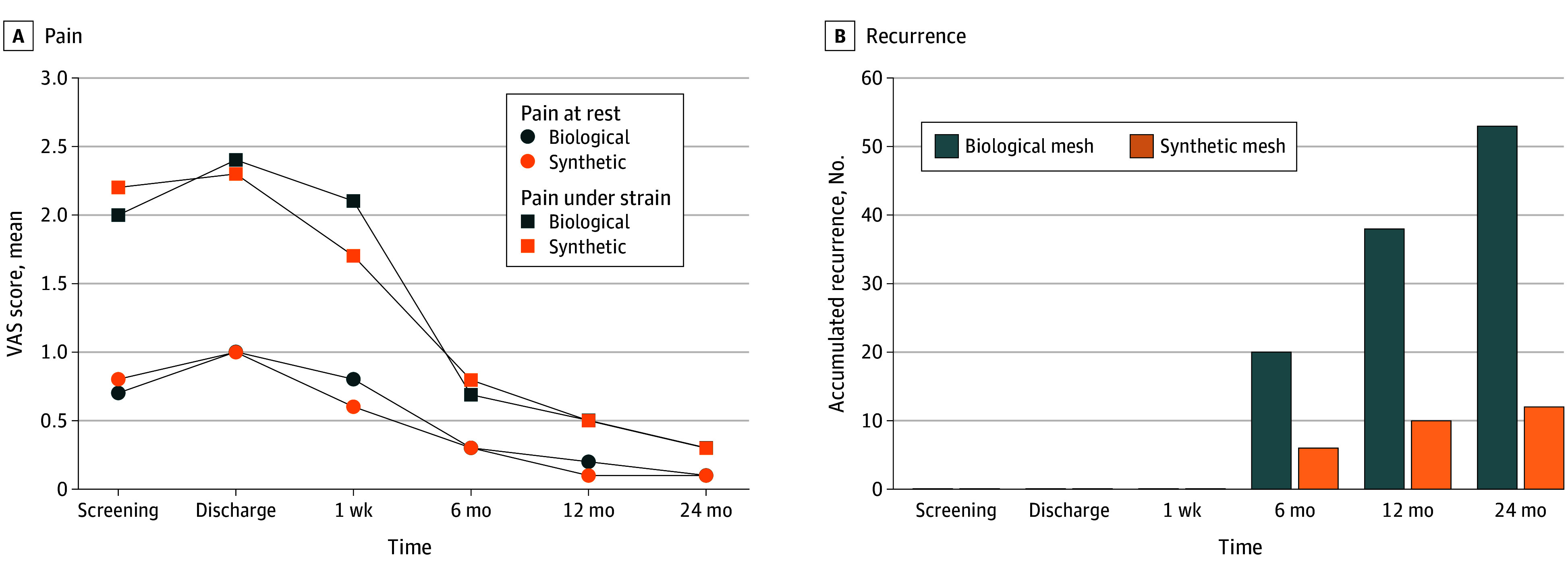
Pain and Recurrence as Primary Outcomes Following Implantation of Biological vs Synthetic Meshes A, Mean visual analog scale (VAS) score for pain at rest and pain under strain with biological vs synthetic mesh. Pain intensity at 6 months was a primary outcome of the study. B, Cumulative number of recurrences. The 2-year recurrence rate was a primary outcome of the study.

Six months after surgery, there were no significant differences in pain intensity between biological and synthetic mesh augmentations. The mean (SD) VAS pain score at rest was 0.3 (0.9) for both materials (*P* = .76) and was 0.7 (1.6) for biological meshes under strain compared with 0.8 (1.6) for synthetic meshes under strain (*P* = .08). The median (IQR) VAS pain score at rest was 0 (0-0) for both materials. Under strain, the median (IQR) VAS pain score remained 0 (0-0) for biological mesh and was 0 (0-1) for synthetic mesh (Table 2 and Figure 2).

**Table 2.** Primary and Secondary Outcomes^a^

Outcome	Patients, No.	Biological mesh	Synthetic mesh	*P* value
**Primary**				
Accumulated recurrence rate at 24 mo, No. (%)	472	53 (11.2)	12 (2.5)	<.001
Pain VAS score at 6 mo, mean (SD)				
At rest	491	0.3 (0.9)	0.3 (0.9)	.76
Under strain	491	0.7 (1.6)	0.8 (1.6)	.08
**Secondary**				
Accumulated recurrence rate, No. (%)				
6 mo	472	20 (4.2)	6 (1.3)	.02
12 mo	472	38 (8.1)	10 (2.1)	<.001
Pain VAS score, mean (SD)				
At rest				
Screening	487	0.7 (1.5)	0.8 (1.6)	.56
Discharge	489	1.0 (1.4)	1.0 (1.3)	.12
1 wk	489	0.8 (1.3)	0.6 (1.2)	.001
12 mo	Biological mesh, 425; synthetic mesh, 442	0.2 (0.7)	0.1 (0.5)	.04
24 mo	Biological mesh, 382; synthetic mesh, 409	0.1 (0.6)	0.1 (0.4)	.31
Under strain				
Screening	487	2.0 (2.4)	2.2 (2.5)	.20
Discharge	489	2.4 (2.0)	2.3 (1.9)	.09
1 wk	489	2.1 (2.1)	1.7 (1.9)	<.001
12 mo	Biological mesh, 425; synthetic mesh, 442	0.5 (1.4)	0.5 (1.2)	.54
24 mo	Biological mesh, 382; synthetic mesh, 409	0.3 (1.1)	0.3 (1.0)	.82
Seroma				
Discharge, No.	491	27	24	NA
1 wk, No.	491	155	95	NA
6 mo, No.	491	13	8	NA
Diagnosed at least once, No. (%)	491	164 (33.4)	106 (21.6)	<.001
Hematoma				
Discharge, No.	491	18	11	NA
1 wk, No.	491	60	50	NA
6 mo, No.	491	6	6	NA
Diagnosed at least once, No. (%)	491	72 (14.7)	59 (12.0)	.16

Abbreviations: NA, not applicable; VAS, visual analog scale.

^a^ Complete follow-up data are available for 472 patients at the primary end points. For 19 patients, follow-up after discharge was incomplete and the last-observation-carried-forward principle was applied for pain intensity 6 months after surgery. Patients with a recurrence were excluded from further pain analysis after diagnosis of recurrence. Loss to follow-up 12 and 24 months after surgery was not completed by the last-observation-carried-forward principle.

At least 1 follow-up examination after 6, 12, or 24 months was required to evaluate for recurrence. Patients without a follow-up examination were excluded from the analysis. Five patients had no follow-up after discharge, a further 14 patients had their latest follow-up examination 1 week after surgery. Excluding these 19 cases, 472 patients remained for analysis.

Among the 472 patients, 63 (13.3%) were diagnosed with a recurrence, including 2 with bilateral recurrences. Of the 65 recurrences, 53 (11.2%) occurred on the side with a biological mesh and 12 (2.5%) on the side with a synthetic mesh (*P* < .001) (Table 2 and Figure 2). The difference of 8.7% (95% CI, 5.5%-11.9%) exceeded the predefined difference of 3% for equivalence. Thus, significantly more recurrences occurred following biological mesh implantation.

In the 5 cases where the mesh material was not implanted as randomized, 1 patient had a recurrence on the side that received synthetic mesh repair, contrary to randomization. According to the ITT analysis, this recurrence was assigned to the biological mesh group. An as-treated evaluation would have only a marginal effect on the difference, with recurrence rates of 11.0% for the biological mesh group and 2.8% for the synthetic mesh group.

### Intensity of Pain at Different Time Points

At screening, pain intensity was similar in both groups (Figure 2 and Table 2). At discharge, patients reported the most severe pain in comparison with the other study visits: there were no significant differences between the mesh materials, with a mean (SD) VAS score of 2.4 (2.0) for biological mesh under strain vs 2.3 (1.9) for synthetic mesh under strain. One week after surgery, there were significant differences in both pain at rest (mean [SD] VAS score, 0.8 [1.3] with biological mesh vs 0.6 [1.2] with synthetic mesh; *P* = .001) and under strain (mean [SD] VAS score, 2.1 [2.1] with biological mesh vs 1.7 [1.9] with synthetic mesh; *P* < .001), with the biological implants associated with more pain than the synthetic meshes.

### Recurrence at 1 Year

Twelve months after surgery, 38 recurrences (8.1%) were reported for the biological mesh group compared with 10 (2.1%) in the synthetic mesh group (*P* < .001) (Figure 2 and Table 2). Again, the 3% difference, which is considered clinically comparable, falls outside the 95% CI.

### Predictors for Recurrence

Multivariate analysis revealed 2 significant factors associated with the development of recurrence: the use of biological mesh material and treatment at a low-volume center, defined as fewer than 50 patients included in the trial. Older patient age and larger hernia size had only minimal effects (Table 3). Mesh fixation, sex, laparoendoscopic technique, and the presence of hematoma did not influence the development of recurrence.

**Table 3.** Multivariate Analysis for Recurrence^a^

Variable	OR (95% CI)	*P* value
Age ≥60 y	1.51 (0.87-2.61)	.14
Female	0.86 (0.29-2.56)	.78
Lateral	0.66 (0.39-1.12)	.12
Biological mesh	4.53 (2.42-8.49)	<.001
Fixation	1.12 (0.66-1.90)	.67
TAPP	0.89 (0.51-1.58)	.70
High-volume center	0.48 (0.28-0.84)	.01
Seroma	1.58 (0.90-2.78)	.11
Hematoma	0.998 (0.49-2.05)	>.99
Size of hernia		
Grade 2	1.21 (0.59-2.49)	.60
Grade 3	1.71 (0.75-3.88)	.20

Abbreviations: OR, odds ratio; TAPP, transabdominal preperitoneal plasty.

^a^ Patients with follow-up at least 6 months after surgery were included in the multivariate analysis. Both patient factors (eg, age) and hernia factors (eg, size, seroma) were considered as possible predictors for the development of a recurrence.

In both the biological mesh group and the synthetic mesh group, patients with recurrence were older than those without recurrence (biological mesh group: mean [SD] age, 61.6 [10.8] vs 57.6 [14.1] years, respectively; synthetic mesh group: mean [SD] age, 60.1 [15.7] vs 58.0 [14.0] years, respectively) (eTable 4 in Supplement 3). Biological meshes from 3 different manufacturers were used. Recurrences were reported for all 3 biological meshes, without significant differences between the manufacturers.

### Incidence of Seromas and Hematomas

Within the first 6 months after surgery, 322 seromas were reported cumulatively across the 3 follow-up visits. Most seromas (n = 250) were observed 1 week after surgery, and no seromas were reported later than 6 months. Some seromas were documented multiple times during the 3 follow-up visits. In 164 patients (33.4%), 1 seroma had been observed at least once on the side with biological mesh implantation, compared with 106 patients (21.6%) after synthetic mesh augmentation. The difference between the 2 materials was statistically significant (*P* < .001).

A hematoma was diagnosed at least once in 72 patients (14.7%) in the biological mesh group vs 59 (12.0%) in the synthetic mesh group. This difference was not statistically significant (*P* = .16) (eTable 5 in Supplement 3).

### Surgical Site Infections

Until discharge, 1 patient developed systemic infection. One week after surgery, a urinary tract infection was reported for 1 patient, and a local infection at the former trocar incision site was reported for 2 patients. After 6 months, another trocar incision site infection and 1 inguinal surgical site infection occurred. No infections were reported beyond 6 months.

### Patient Satisfaction

At each postoperative visit, patients were asked separately about both sides whether they experienced paresthesia or foreign-body sensation and whether they were more satisfied with one side than the other. Postoperative paresthesia was reported in less than 5% of the repairs, with no significant differences between mesh materials at any time (eTable 6 in Supplement 3). Only at 1 week postoperatively, but not at any other study visits, foreign-body sensation was significantly more common with biological implants (49 patients [10.4%]) compared with synthetic meshes (30 patients [6.3%]) (*P* = .03) (eTable 7 in Supplement 3).

Patient satisfaction did not differ significantly between mesh materials, except at 1 week postoperatively, when 150 patients (31.7%) preferred synthetic mesh vs 90 patients (19.0%) preferring biological mesh (*P* < .001) (eTable 8 in Supplement 3).

## Discussion

To our knowledge, the BIOLAP randomized clinical trial is the first hernia trial with a self-controlled design. This minimizes selection bias as it allows the inclusion of a broad study population, which is confirmed by the close similarity to the patients’ cohort with bilateral hernias in the German hernia registry.^3,19^ External confounding factors, such as surgical technique and surgeon experience, as well as internal factors, like preexisting conditions, affect both study arms equally and hence do not influence the results. The design allows for an ideal comparison of subjective ratings, like pain or preference. With the exception of the 2 mesh dislocations, the typical complications of laparoendoscopic hernia surgery occurred.^3,19^ The median length of hospital stay of 2 nights is identical to the published data from the German hernia register Herniamed^19^ and most likely is a result of financial incentives by the reimbursement system in Germany at that time. It can therefore be stated that the patient population of this study reflects real-life conditions and that results are transferable to clinical application.

The idea of a resorbable, yet stabilizing biological implant alternative to standard hernia mesh material is promising. Because of the absence of a lifelong remaining synthetic mass, chronic inguinal pain was expected to decrease. Primary as well as secondary end points showed that biological mesh implantation did not reduce postoperative pain, with both mesh materials yielding comparably low VAS scores 6 months postoperatively. The significantly increased seroma rate, pain intensity, and foreign-body sensation as well as reduced patient satisfaction 1 week after surgery in the biological mesh arm needs to be further investigated and could be an indicator for an increased transient inflammatory response in the postoperative period that resolves later.^20^

The more than 4-fold higher recurrence rate observed with resorbable biological implants clearly demonstrates the inferiority of this material in terms of stability. After just 1 year, treatment with biological meshes was significantly inferior to repair with permanent synthetic meshes. If tissue remodeling is indeed a valid phenomenon, it appears to be inferior to mechanical wall reinforcement provided by a remaining stabilizing implant—whether in terms of a less effective mechanism of action, shorter duration of effect, or both. There was no evidence for a stronger influence of patient age on recurrences after biological mesh repair compared with synthetic mesh repair. If there is a lasting cell invasion–based stabilizing mechanism, it appears age independent. Because of the small number of recurrences following the use of synthetic mesh, this study is not powered to evaluate and compare other predictors for hernia recurrence between both mesh materials.

Our data provide no evidence that biological mesh is a preferable treatment for any patient subgroup who is also eligible for a permanent implant. Furthermore, the data show that the choice of implant material was such a significant predictor of recurrence that it outweighed all other technical and individual influences. The experience of the treating institution appears to be the only other important factor for recurrence development in our data. Surgery in a high-volume center is associated with fewer recurrences, reflecting the expertise of not only the surgeon but the whole medical and nursing team. This has also been shown in previous studies.^21^

Our study results are inconsistent with those of a recently published randomized clinical trial by Xue et al^22^ about biologics and lightweight synthetic meshes for laparoscopic inguinal hernia repair that reported comparable effectiveness and fewer postoperative complications for the biological mesh. The results regarding the recurrence rate, postoperative pain intensity, and frequency of seroma occurrence in previous studies should be interpreted with caution, as these were mostly conducted monocentrically with a singular product, small numbers of patients, and short follow-up periods and without the comparability provided by the self-controlled study design.^12,23^

Biosynthetic meshes are a relatively new type of nonpermanent implant that are absorbed more slowly (up to 36 months) than biological meshes and are intended to combine the advantages of biological and synthetic material. There is increasing evidence that this kind of mesh may be improved in terms of recurrence rate.^24^ This remains to be confirmed by reasonably sized randomized clinical trials, preferably with a self-controlled design like BIOLAP.

The data show that permanent synthetic meshes were preferable to current resorbable biological meshes. It remains to be answered how much the surgical preparation contributes to the prevention of a recurrence and whether there is any lasting effect from the implantation of the biological matrix.

### Strengths and Limitations

To our knowledge, this is the only study in hernia surgery to use a self-controlled design. The inclusion and exclusion criteria were kept to a minimum, allowing for the inclusion of a broad patient population that reflects real-world clinical conditions.

Several limitations should be noted. First, even though the participating centers were allowed to use products of their choice within the given criteria, only 3 different biological meshes by 3 manufactures were used. This was likely due to the significantly higher costs associated with other available products. Second, although the study was conducted across multiple centers, recruitment rates varied significantly—3 centers had high recruitment, while several others had very low participation. This imbalance limits the comparability between centers. Third, the study included only patients with bilateral hernias, who may have had different collagen metabolism and other influencing factors compared with individuals with unilateral hernias.

## Conclusions

This study found that biological mesh implantation in laparoendoscopic inguinal hernia repair did not reduce postoperative pain but significantly increased the risk for hernia recurrence and seroma compared with standard synthetic meshes. These findings do not support the routine use of biological meshes in laparoendoscopic inguinal hernia repair.

## Notes

Supplement 1. Trial Protocol

Supplement 2. Statistical Analysis Plan

Supplement 3. **eAppendix.** List of Study Investigators and Sites **eTable 1.** Hernia Subtypes **eTable 2.** Postoperative Complications **eTable 3.** Biological and Synthetic Meshes Used in the BIOLAP Trial **eTable 4.** Recurrence and Patient Age **eTable 5.** Seroma and Hematoma **eTable 6.** Postoperative Paresthesia **eTable 7.** Postoperative Foreign Body Sensation **eTable 8.** Postoperative Patient Satisfaction

Supplement 4. Data Sharing Statement

## References

[1] HerniaSurge Group. International guidelines for groin hernia management. Hernia. 2018;22(1):1-165. doi:10.1007/s10029-017-1668-x 29330835 PMC5809582

[2] Primatesta P, Goldacre MJ. Inguinal hernia repair: incidence of elective and emergency surgery, readmission and mortality. Int J Epidemiol. 1996;25(4):835-839. doi:10.1093/ije/25.4.835 8921464

[3] Jacob DA, Hackl JA, Bittner R, Kraft B, Köckerling F. Perioperative outcome of unilateral versus bilateral inguinal hernia repairs in TAPP technique: analysis of 15,176 cases from the Herniamed Registry. Surg Endosc. 2015;29(12):3733-3740. doi:10.1007/s00464-015-4146-5 25786904 PMC4648949

[4] Sayad P, Abdo Z, Cacchione R, Ferzli G. Incidence of incipient contralateral hernia during laparoscopic hernia repair. Surg Endosc. 2000;14(6):543-545. doi:10.1007/s004640000101 10890962

[5] Karthikesalingam A, Markar SR, Holt PJ, Praseedom RK. Meta-analysis of randomized controlled trials comparing laparoscopic with open mesh repair of recurrent inguinal hernia. Br J Surg. 2010;97(1):4-11. doi:10.1002/bjs.6902 20013926

[6] Aiolfi A, Cavalli M, Del Ferraro S, . Total extraperitoneal (TEP) versus laparoscopic transabdominal preperitoneal (TAPP) hernioplasty: systematic review and trial sequential analysis of randomized controlled trials. Hernia. 2021;25(5):1147-1157. doi:10.1007/s10029-021-02407-7 33851270 PMC8514389

[7] Köckerling F, Krüger C, Gagarkin I, . What is the outcome of re-recurrent vs recurrent inguinal hernia repairs? an analysis of 16,206 patients from the Herniamed Registry. Hernia. 2020;24(4):811-819. doi:10.1007/s10029-020-02138-1 32086633 PMC7395905

[8] Poobalan AS, Bruce J, Smith WC, King PM, Krukowski ZH, Chambers WA. A review of chronic pain after inguinal herniorrhaphy. Clin J Pain. 2003;19(1):48-54. doi:10.1097/00002508-200301000-00006 12514456

[9] Haapaniemi S, Gunnarsson U, Nordin P, Nilsson E. Reoperation after recurrent groin hernia repair. Ann Surg. 2001;234(1):122-126. doi:10.1097/00000658-200107000-00018 11420492 PMC1421957

[10] Nienhuijs S, Staal E, Strobbe L, Rosman C, Groenewoud H, Bleichrodt R. Chronic pain after mesh repair of inguinal hernia: a systematic review. Am J Surg. 2007;194(3):394-400. doi:10.1016/j.amjsurg.2007.02.012 17693290

[11] Bochicchio GV, Jain A, McGonigal K, . Biologic vs synthetic inguinal hernia repair: 1-year results of a randomized double-blinded trial. J Am Coll Surg. 2014;218(4):751-757. doi:10.1016/j.jamcollsurg.2014.01.043 24655865

[12] Sun L, Chen J, Li J, Shen Y. Randomized and comparative clinical trial of bovine mesh versus polypropylene mesh in the repair of inguinal hernias. Surg Laparosc Endosc Percutan Tech. 2020;30(1):26-29. doi:10.1097/SLE.0000000000000744 31876883

[13] Ravo B, Falasco G. Pure tissue inguinal hernia repair with the use of biological mesh: a 10-year follows up: a prospective study. Hernia. 2020;24(1):121-126. doi:10.1007/s10029-019-01976-y 31111323

[14] Louis TA, Lavori PW, Bailar JC III, Polansky M. Crossover and self-controlled designs in clinical research. N Engl J Med. 1984;310(1):24-31. doi:10.1056/NEJM198401053100106 6689736

[15] Sauerland S, Lefering R, Bayer-Sandow T, Brüser P, Neugebauer EA. Fingers, hands or patients? the concept of independent observations. J Hand Surg Br. 2003;28(2):102-105. doi:10.1016/S0266-7681(02)00360-1 12631478

[16] Seefeldt CS, Meyer JS, Knievel J, . BIOLAP: biological versus synthetic mesh in laparo-endoscopic inguinal hernia repair: study protocol for a randomized, multicenter, self-controlled clinical trial. Trials. 2019;20(1):55. doi:10.1186/s13063-018-3122-5 30651127 PMC6335692

[17] Schulz KF, Altman DG, Moher D; CONSORT Group. CONSORT 2010 statement: updated guidelines for reporting parallel group randomised trials. BMJ. 2010;340:c332. doi:10.1136/bmj.c332 20332509 PMC2844940

[18] Miserez M, Alexandre JH, Campanelli G, . The European hernia society groin hernia classification: simple and easy to remember. Hernia. 2007;11(2):113-116. doi:10.1007/s10029-007-0198-3 17353992

[19] Köckerling F, Adolf D, Lorenz R, . Perioperative outcome in groin hernia repair: what are the most important influencing factors? Hernia. 2022;26(1):201-215. doi:10.1007/s10029-021-02417-5 33895891

[20] Kokotovic D, Burcharth J, Helgstrand F, Gögenur I. Systemic inflammatory response after hernia repair: a systematic review. Langenbecks Arch Surg. 2017;402(7):1023-1037. doi:10.1007/s00423-017-1618-1 28831565

[21] Maneck M, Köckerling F, Fahlenbrach C, . Hospital volume and outcome in inguinal hernia repair: analysis of routine data of 133,449 patients. Hernia. 2020;24(4):747-757. doi:10.1007/s10029-019-02091-8 31786700 PMC7395912

[22] Xue P, Yue F, Li S, . A multicenter randomized controlled trial comparing short- and medium-term outcomes of novel biologics and lightweight synthetic mesh for laparoscopic inguinal hernia repair. Hernia. 2024;28(4):1337-1344. doi:10.1007/s10029-024-03046-4 38902558

[23] Cuihong J, Deyu T, Yingmo S. Outcomes of porcine small intestinal submucosa mesh compared to polypropylene mesh in laparoscopic transabdominal preperitoneal inguinal hernia repair: a retrospective cohort study. Surg Endosc. 2025;39(2):952-959. doi:10.1007/s00464-024-11355-z 39653858

[24] Al-Bustami IS, Clements T, Ferguson D, Harmouch A, Olavarria OA, Holihan JL. Biosynthetic mesh in hernia repair: a systematic review and meta-analysis. Int J Abdom Wall Hernia Surg. 2024;7(2):55-66. doi:10.4103/ijawhs.ijawhs_99_23

