# Supplementary material for: Biological vs Synthetic Mesh in Laparoendoscopic Inguinal Hernia Repair: The BIOLAP Randomized Clinical Trial
Source: JAMA Surg. 2025 Oct 8;160(12):1309–16. doi: 10.1001/jamasurg.2025.4071 (PMC12509081; doi:10.1001/jamasurg.2025.4071)
Supplement: Supplement 1. — Trial Protocol [file jamasurg-e254071-s001.pdf]

STUDY PROTOCOL

BIOLAP: Biological versus synthetic mesh in laparoscopic hernia repair - a randomised multicentre, prospective, self-controlled clinical trial -

Sponsor:
Witten/Herdecke University
represented by
ZKS-UW/H
Dr. Wolfgang Eglmeier
Alfred-Herrhausen-Str. 50
58448 Witten
Tel.: +49 (0)2302 926-708
Wolfgang.eglmeier@uni-wh.de

Head of the clinical trial:
Prof. Dr. med. M. Heiss
Viszeral-, Gefäß- und Transplantationschirurgie, Klinikum Köln Merheim
Ostmerheimer Str. 200, 51109 Köln
+49(0) 221 89073770
HeissM@kliniken-koeln.de
(member of the Fakulty of Health of Witten/Herdecke University)

Study Protocol Code: BIOLAP

Version V05, 05.03.2020

The information in this protocol is strictly confidential. It is intended only for the information of the sponsor, the investigators, the study staff, the ethics committee, the authorities and the patients. This protocol may not be passed on to third parties without the consent of the sponsor or the head of the clinical trial (LKP).

I. Unterschriften

Dr. Wolfgang Eglmeier<br>Leiter ZKS-UW/H<br>In Vertretung des Sponsors	Unterschrift	06.04.20<br>Datum
Prof. Dr. Markus Heiss<br>Leiter der klinischen Prüfung/<br>Medizinischer Ansprechpartner des Sponsors	Unterschrift	31.3.20<br>Datum
Prof. Dr. Rolf Lefering<br>Institut für Forschung in der operativen Medizin	31.3.20<br>Unterschrift	31.3.20<br>Datum
Name<br>Lokaler Prüfer	Unterschrift	Datum

II. Synopsis

Sponsor:	Witten/Herdecke University<br>Alfred-Herrhausen-Straße 50<br>58448 Witten<br>represented by:<br>Centre for Clinical Studies at Witten/Herdecke University<br>Alfred-Herrhausen-Straße 50<br>58448 Witten
Head of the clinical trial:	Prof. Dr. med. M. Heiss<br>Viszeral-, Gefäß- und Transplantationschirurgie, Klinikum Köln Merheim, Ostmerheimer Str. 200, 51109 Köln<br>+49(0) 221 89073770<br>HeissM@kliniken-koeln.de
Title of the clinical trial:	BIOLAP: Biological versus synthetic mesh in laparoscopic hernia repair - a randomised multicentre, prospective, self-controlled clinical trial
Indication:	Bilateral primary inguinal hernias
Type of examination, study design/ methodology:	Prospective, intrinsically controlled, two-arm comparative study of two CE-certified medical devices in the intended indication with regard to efficacy
Number of patients:	496 patients with bilateral inguinal hernia → 992 hernias
Primary study objective:	It should be shown that the use of biological mesh material for laparoscopic hernia treatment results in significantly less postoperative pain than the use of synthetic mesh material, without an increased recurrence rate

Outcome:	Primary Outcome:<br>• Pain reduction after 6 months measured with a visual analogue scale (VAS 0-10)<br>• Recurrence rate after 2 years<br>Secondary Outcome:<br>• Frequency and intensity of pain at other time points (one week and one year after surgery)<br>• Recurrence rate after one year<br>• Local infection of the surgical site<br>• Mesh dislocation<br>• Haematoma<br>• Seroma<br>• Patient satisfaction (overall result, foreign body sensation, paraesthesia, one week and one year after surgery)
Evaluation criteria:	Effektiveness:<br>• The expected advantage of the biological mesh is 0.5 points on the 0-10 VAS.<br>• An assumed recurrence rate after 2 years of 5 % for the synthetic mesh material contrasts with a recurrence rate of ≤ 8 % for the biological mesh material.<br>Security:<br>• Number and type of complications such as re-interventions or replacement of the mesh

Diagnosis and main inclusion criteria:	Diagnosis/main inclusion criterion:<br>• Patients with bilateral primary inguinal hernias<br>Further inclusion criteria:<br>• Patient is suitable for laparoscopic surgery<br><br>Main exclusion criteria:<br>• Recurrent Hernia<br>• Incarcerated Hernia<br>• Presence of an acute systemic infection
Test product:<br>Type of application:	Not two specific products but two types of materials are compared. Each study centre must use commercially available mesh material. However, the following requirements apply:<br>• CE-certified meshes<br>• The synthetic mesh materials should be made of polypropylene, polyester or PVDF, have large pores and be lightweight (< 100 g/m^2).<br>• The biological mesh material should consist of a perforated, non-crosslinked, acellular and collagenous matrix.<br>• The meshes should have a minimum size of 10x15 cm<br>• The meshes can be fixed with adhesive
Comparative therapy, dosage and method of application:	Both mesh materials are used for each patient and compared directly with each other (self-controlled design). Other procedures/materials are not usual for this indication. The usual laparoscopic implantation in hernia repair is performed according to IEHS guidelines.
Duration of therapy:	The duration of treatment corresponds to the clinical routine and the procedure itself takes about 1 hour. Both hernias are treated in one procedure.

Schedule:

Inclusion of the first patient<br>(first patient first visit, FPFV):	01.08.2017
Inclusion of last patient (LPFV):	31.07.2020
End of examination of the last patient (LPLV):	31.07.2022
Integrated final report:	31.07.2023

Statistician:

Prof. Dr. Rolf Lefering
Institute for Research in Operative Medicine
Witten/Herdecke University
Ostmerheimer Straße 200, Haus 38
51109 Köln

Statistical methods:	Hernias, not patients, are randomised. In each patient, one hernia is treated with a synthetic mesh and the hernia on the other side with biological mesh material.

Primary endpoint efficacy:

All endpoints are analysed in the intention-to-treat population. In the absence of follow-up examinations, the LOCF (Last Observation Carried Forward) principle is used. Two primary endpoints are analysed (recurrence and pain); the p-value is adjusted according to Bonferroni. Pain reduction is determined separately for each side. The pain analysis is performed using the Wilcoxon signed-rank test for paired samples (alpha 0.025, two-sided). The evaluation of the recurrence rate requires a minimum observation period of 2 years. The difference in recurrence rates is expressed as a 95 % confidence interval (one-sided).

Safety:

Complications are compared with the McNemar test for the event rate in paired data.

Secondary endpoints:

Early recurrence rates (up to one year post-op) are analysed descriptively using Kaplan-Meier. Pain intensity and frequency at all recorded time points are analysed with a non-parametric rank test.

GCP confirmity:	This trial will be conducted in accordance with the current version of the protocol, the internationally recognised guideline Good Clinical Practice (ICH-GCP) and Good Clinical Practice for the Investigation of Medical Devices (DIN EN ISO 14155), including the archiving of essential documents, but with an adapted scope due to the study design (CE-certified medical devices in indication § 23b MPG).

Financing: Funding is provided by the German Research Foundation (grant number HE 1586/5-1)

III. Table of contents

I. Signatures 2

II. Synopsis 3

III. Table of contents 9

III.a) List of tables 12

III.b) List of illustrations 12

IV. List of abbreviations 13

1. Introcution 14

2. Aims of the clinical trial 15

2.1. Rationale of the clinical trial 15

2.2. Primary Outcome 16

2.3. Secondary outcome 16

3. Organisational structure 17

3.1. Sponsor 17

3.2. Head of the clinical trial 17

3.3. Statistics/Date management 17

3.4. Data Safety Monitoring Committee (DSMC) 17

3.5. Test laboratories and other technical facilities 18

3.6. Central organisational units 18

3.7. Investigator and study sites 19

3.8. Financing 19

4. Study realisation 20

4.1. General study design 20

4.1.1. Timetable 20

4.2. Discussion of the study design 21

4.3. Selection of the study population 22

4.3.1. Inclusion criteria 22

4.3.2. Exclusion criteria 22

4.4. Subsequent exclusion of study participants 23

4.4.1. Procedure in the event of premature termination of treatment in the clinical trial 23

4.5. Closure of trial centres/early termination of the clinical trial 24

4.6. Treatments 24

4.6.1. Treatments used 24

4.6.2. Description of the medical device 25

4.6.2.1. Labelling of the medical device 25

4.6.2.2. Storage of the medical device 25

4.6.3. Method for assigning patients to the treatment groups and blinding 25

4.6.4. Previous therapy and concomitant therapy 26

4.6.4.1. Alternative therapy in an emergency 26

4.6.5. Further treatment after the end of the clinical trial 26

4.7. Efficacy and safety parameters 26

4.7.1. Measurement of efficacy and safety parameters 26

4.7.1.1. Primary and secondary outcomes 26

4.7.1.2. Further outcomes 27

4.7.1.3. Safety analysis 27

4.7.1.4. Description of the individual visits 27

4.7.2. Appropriateness of the measurement methods 30

4.8. Ensuring data quality 30

4.8.1. Monitoring 30

4.8.2. Audits/Inspections 31

4.9. Documentation 32

4.9.1. Data management 32

4.9.1.1. CRF: Demographic data and preoperative data 33

4.9.1.2. Preoperative preparation (D-3 bis D-1) 34

4.9.1.3. CRF: Operation data 34

4.9.1.4. CRF: hospital course/discharge (D2-D4) 35

4.9.1.5. CRF: Follow-up 2 years by centres with consultations: 35

4.9.2. Archiving 36

5. Ethical and regulatory aspects 37

5.1. Independent ethics committees 37

5.2. Ethical conduct of the clinical trial 37

5.2.1. Legal provisions, guidelines and standards taken into account 37

5.3. Notifications to authorities, authorisations and registration 37

5.4. Information and consent of the test subjects 38

5.5. Clinical trials insurance 38

5.6. Data protection 39

6. Statistical methods and determination of the number of cases 40

6.1. Statistical and analytical plan 40

6.1.1. Study populations 40

6.1.2. Description of the patient collective 40

6.1.3. Primary outcomes 41

6.1.4. Secondary outcomes 41

6.1.5. Determination of the number of cases 41

7. Use of the data and publication 43

7.1. Reports 43

7.1.1. Interim reports 43

7.1.2. Final report 43

7.2. Publication 43

8. Changes to the study protocol 45

9. Literature 46

10. Attachments 48

10.1. Participating trial sites and principal investigators 48

10.2. Patient information and declaration of consent 48

III.a) List of tables

Table 1: Schedule of the trial 20

Table 2 Flow Chart 27

III.b) List of illustrations

Figure 1: Prodecure of the clinical trial 21

IV. List of abbreviations

Abbreviation	Meaning
ADE	Adverse Device Effect
ASADE	Anticipated Serious Adverse Device Effect
CRF	Case Report Form
DALY	Disease-adjusted life years
DSMC	Data Safety Monitoring Committee
IFOM	Institute for Research on operative medicine
IFU	Instructions For Use
LKP	Leiter der klinischen Prüfung (Principal Coordinating Investigator)
SADE	Serious Adverse Device Effect
SUSADE	Suspected Unexpected Serious Adverse Device Effect
TAPP	transabdominal preperitoneal plasty
TEP	total extraperitoneal plasty
USADE	Unanticipated Serious Adverse Device Effect
ZKS-UW/H	Zentrum für klinische Studien der Universität Witten/Herdecke/Center for clinical trials

1. Introcution

Inguinal hernia repair is one of the most common surgical procedures, with more than 20 million being treated each year (6). In the USA alone, approximately 4.5 million people are affected (1). Bilateral inguinal hernias occur in approximately 30% of adult cases (2). The risk of developing an inguinal hernia in the course of a lifetime is 27% for men and 3% for women (3). More than 700 deaths each year in England and Wales are attributable to abdominal hernias, a higher number than for gallstones, pancreatitis or inflammatory bowel disease (4). Abdominal hernias are generally considered to be amenable to surgical repair, so this high mortality rate is generally considered preventable (5). A 2010 study by the Global Burden of Disease project found that 11 DALYs per 100,000 population per year are attributable to inguinal hernias (7).

Inguinal hernias can be treated surgically in various ways: open procedures, open procedures with mesh inserts and laparoscopic procedures with mesh inserts (8). Laparoscopic procedures have proven their worth as they provide very good results and also result in less post-operative pain and wound infections (9). However, hernia recurrence is a clinical problem, with a recurrence rate of 1-10% (10) and the occurrence of chronic pain after hernia repair is also problematic with a frequency of 11-54% (11, 12).

For laparoscopic hernia repair, two standard procedures have been established as equivalent in terms of recurrence rate and occurrence of pain: TAPP (transabdominal preperitoneal plasty) and TEP (total extraperitoneal plasty). Nowadays, it common practice to use synthetic mesh material for the plastic. However, there is increasing evidence that biological mesh material offers advantages in terms of the occurrence of chronic pain due to the different postoperative remodelling without disadvantages of a lifelong artificial implant (13).

To date, however, there have been no studies that have specifically investigated whether the presumed advantage of biological mesh material can also be empirically proven.

2. Aims of the clinical trial

The aim is to show that the use of biological mesh material for the laparoscopic treatment of inguinal hernias causes significantly less pain postoperatively than the use of synthetic mesh material (superiority), without an increased recurrence rate (non-inferiority).

2.1. Rationale of the clinical trial

A literature search in the PubMed database using the search terms biological mesh (biomesh) and inguinal hernia (groin hernia) found only one study comparing biological mesh material in open hernia repair and also reporting only a 1-year follow-up (13). This study showed initial indications of a reduction in pain when using the biological mesh material, while the recurrence rate appeared to depend more on the surgeon's experience. The only study dealing with the use of biological mesh material in the TAPP procedure is a retrospective case series of 11 patients, which confirms that the biological material is very suitable for laparoscopic hernia repair. However, there is no self-controlled, prospective, multicentre study as envisaged in the present case. The result of the literature search was also confirmed in a recently published review article (15).

Since, despite many indications of the advantages of using biological mesh material for hernia repair and its frequent use in everyday clinical practice, there is no reliable evidence in favour of using biological rather than synthetic mesh material, we believe that a prospective study is necessary. If it can be proven that biological mesh material for hernia repair has no disadvantage in terms of recurrence rate and pain intensity/frequency, this could be a milestone in hernia repair and make biological mesh material the standard implant for laparoscopic repair of inguinal hernias.

The central element of the study design is self-control. Only patients with bilateral hernias are included and a biological mesh is then implanted on one side of each patient and the synthetic mesh material is used on the other side. Which side is treated with which material is decided in advance by randomisation. Each patient is therefore their own control and allows an ideal comparison of the two materials without additional confounding variables.

Due to the limited data on the use of biomeshes in hernia surgery, the study is to be extended to up to ten years of follow-up after completion of the two-year observation phase. Participation in the long-term data collection is voluntary for the study participants, all study participants can separately consent to further participation in the study. During the further follow-up, each patient will remain in the study until bilateral recurrence, up to a maximum of ten years after surgery.

The study endpoints remain the same. Long-term observation makes sense, as no studies with a sufficiently high number of cases are known to date that observe the use of biomeshes over a longer period of time. In addition, many recurrences only occur after more than two years [17].

2.2. Primary Outcome

This study has two primary endpoints: Pain intensity after 6 months, measured with the visual analogue scale VAS (0-10) and the recurrence rate after 2 years.

Pain intensity is tested for difference, while non-inferiority is tested for recurrence rate.

2.3. Secondary outcome

- Intensity of pain at other time points (see table Flow Chart)
- Recurrence rates within one year after surgery
- Complications e.g. infections, mesh dislocations
- Haematoma
- Seroma
- Patient satisfaction (overall result, foreign body sensation, paraesthesia) at various points in time (see table flow chart)

3. Organisational structure

3.1. Sponsor

Sponsor	Witten/Herdecke University<br>Alfred-Herrhausen-Straße 50<br>58448 Witten
represented by:	Dr. Wolfgang Eglmeier, Head of Center for clinical trials of the Witten/Herdecke University<br>Alfred-Herrhausen-Straße 50, 58448 Witten<br>+49(0) 2302 926 708<br>wolfgang.eglmeier@uni-wh.de

3.2. Head of the clinical trial

Head of the clinical trial (LKP)	Prof Dr Markus Heiss<br>Viszeral-, Gefäß- und Transplantationschirurgie,<br>Klinikum Köln Merheim,<br>Ostmerheimer Str. 200, 51109 Köln<br>+49(0) 221 89073770<br>HeissM@kliniken-koeln.de

3.3. Statistics/Date management

Statistician:	Prof. Dr. Rolf Lefering<br>Institute for Research in Operative Medicine<br>Witten/Herdecke University<br>Ostmerheimer Str. 200, Haus 38<br>51109 Köln<br>+49(0) 221 98957-19<br>Rolf.Lefering@uni-wh.de

3.4. Data Safety Monitoring Committee (DSMC)

Complications are documented in the CRF. Those related to the surgical procedure are periodically reviewed by the LKP and the DSMC to determine whether they are (serious) adverse device events (ADEs or SADEs). Members of the DSMC will be:

- Prof. Rudolph Pointner (Ordination Dr. Pointner, Zell am See, Austria)
- Prof. Stefan Sauerland (Institute for Quality and Efficiency in Health Care, Köln, Germany)

3.5. Test laboratories and other technical facilities

The laboratory tests planned as part of this clinical trial are carried out in the clinical laboratories of the participating trial centres.

3.6. Central organisational units

Project Management:	Claudia Simone Seefeldt<br>Visceral-, Vascular- and Transplant Surgery,<br>Klinikum Köln Merheim<br>Ostmerheimer Str. 200<br>51109 Köln<br>+49(0) 221 8907-18500<br>seefeldtsi@kliniken-koeln.de<br><br>together with<br><br>Dr. Judith Knievel<br>Institute for Research in Operative Medicine (IFOM)<br>Witten/Herdecke University<br>Ostmerheimer Str. 200, Haus 38<br>51109 Köln<br>+49(0) 221 98957-24<br>Judith.Knievel@uni-wh.de
Monitoring:	IFOM
Data Management:	IFOM
Coordination DSMC:	Prof. Dr. med. Markus Heiss<br>Visceral-, Vascular- and Transplant Surgery,<br>Klinikum Köln Merheim,<br>Ostmerheimer Str. 200<br>51109 Köln<br>+49(0) 221 8907-3770<br>HeissM@kliniken-koeln.de
Regulatory Support:	Center for clinical trials of the<br>Witten/Herdecke University<br>Alfred-Herrhausen-Straße 50<br>58448 Witten<br>Tel.: +49(0) 2302 926 708<br>Email: zks@uni-wh.de

3.7. Investigator and study sites

The clinical trial will be conducted in a multi-centre setting at 22 trial sites in Germany. If necessary, further qualified trial sites can be included in the clinical trial.

A list of the trial sites with the names of the principal investigators can be found in Annex 10.1. The information on trial sites, principal investigators and other investigators as well as the other trial personnel will be continuously updated in a separate list. The final list will be attached to the final report of the clinical trial.

Requirements for Investigators and study sites

Investigators, monitors and study management must be GCP trained, or will be by the time they are required to fulfil their responsibilities. The trial centres and investigators must have proven expertise in the field of laparoscopic hernia surgery and be able to perform the operation in accordance with the Guidelines for laparoscopic (TAPP) and endoscopic (TEP) treatment of inguinal hernia of the International Endohernia Society (IEHS).

3.8. Financing

Funding is provided by the German Research Foundation (DFG) under the grand number HE 1586/5-1.

4. Study realisation

4.1. General study design

Prospective, intrinsically controlled, two-arm comparative study of CE-certified medical devices in the intended indication with regard to efficacy.

4.1.1. Timetable

Table 1: Schedule of the trial

Inclusion of the first patient (first patient first visit, FPFV):	01.08.2017
Inclusion of last patient (LPFV):	31.07.2020
End of examination of the last patient (LPLV):	31.07.2022
Integrated final report:	31.07.2023

Potential study participants are patients with bilateral inguinal hernias who are identified during routine clinical practice in the participating trial centres (pre-screening). Suitable patients (see also inclusion and exclusion criteria) will be informed about the study by a medical member of the study group. After consent has been given, they are examined for the presence of all inclusion and absence of all exclusion criteria as part of the screening examination.

The treatment of patients then essentially corresponds to the routine laparoscopic repair (TAPP or TEP) of inguinal hernias (in accordance with IEHS guidelines), with the difference that one side is treated with a synthetic mesh material and the other side with biological mesh material. Which side is treated with which material is decided for each patient in advance by randomisation. After discharge, several follow-up examinations are carried out to record the study objectives (see Fig. 1). Discharge usually takes place 1-6 days after surgery. However, depending on the healing process, this can also occur later in individual cases and exceeding the 6 days therefore does not constitute a protocol violation. The reasons for the delayed healing process must be documented and the first follow-up is carried out as an inpatient in this case.

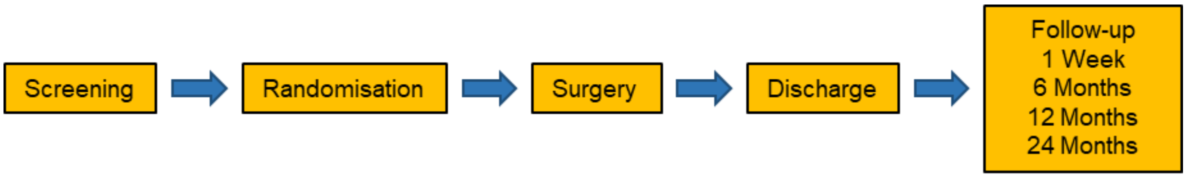

Figure 1: Prodecure of the clinical trial

At the last follow-up visit after two years, the patient can decide whether they would like to take part in further follow-up visits five and ten years after the operation. These further follow-up visits serve to collect additional long-term data and are analysed independently of the original study. If the patient does not consent, the study ends with the follow-up visit "24 months after surgery". After renewed consent, the patient remains in the study until a recurrence occurs on both sides, but for a maximum of ten years.

End of examination

The trial ends with the follow-up visit of the last study participant (LPLV) and the closing of the database.

4.2. Discussion of the study design

The present study is a comparative study designed to show whether biological mesh material has advantages over synthetic mesh material for the repair of inguinal hernias. Both the surgical procedure and the mesh materials used correspond to the standard in clinical routine, the procedure is in accordance with the Guidelines for Laparoscopic (TAPP) and endoscopic (TEP) treatment of inguinal hernia of the IEHS and CE-certified commercial products are used. Only patients with bilateral inguinal hernias are included (approximately 20% of patients scheduled for surgery have bilateral hernias). This allows each patient to serve as a separate control. For this purpose, a decision is made by randomisation before the operation on which side the biological mesh material will be used and on the other side the synthetic mesh material will be used. This allows an ideal comparison between the two mesh materials without additional confounding variables.

4.3. Selection of the study population

The study population is deliberately intended to represent as broad a range of patients as possible who suffer from bilateral inguinal hernias and come from routine clinical practice. Therefore, few restrictions are placed on the inclusion criteria. The exclusion criteria also represent a minimum that is either necessary for safety reasons or interferes with the endpoints.

A specific gender distribution is not required.

4.3.1. Inclusion criteria

- Age at least 18 years
- Written informed consent
- Unrestricted legal capacity
- Primary, bilateral inguinal hernias (if necessary, imaging confirmation by means of sonography/CT/MRI), if necessary, indication if one hernia is larger than the other
- Patient suitable for laparoscopic hernia surgery

4.3.2. Exclusion criteria

- Recurrent hernia
- Incarcerated hernia
- Acute systemic infection
- Expected non-compliance with any study requirement
- Severe comorbidity (ASA ≥ 4)
- Life expectancy < 12 months
- Chemotherapy within the last 4 weeks
- Radiotherapy within the last 2 months
- Known allergy to pig or bovine antigens
- Simultaneous participation in another study that interferes with this study
- Previous participation in this study

- Persons who are in a dependent/employment relationship with the sponsor or investigator
- Placement in an institution on the basis of a court or official order.

4.4. Subsequent exclusion of study participants

If one or more of the following reasons are present, study participants will be excluded from the clinical trial:

- At the request of the study participant (even without giving reasons)
- Based on the decision of an auditor (e.g. after a changed risk analysis)
- Based on the decision of the sponsor (e.g. following a changed risk analysis)
- In case of violation of inclusion or exclusion criteria
- Non-compliance of the study participant

The data collected up to the point of exclusion is used for analysis and the patient must agree to this in the declaration of consent.

A study participant is subsequently excluded from the clinical trial if the laparo-endoscopic treatment of the hernia with a biomesh is not performed (e.g. intraoperative complications, only one-sided hernia diagnosed intraoperatively, other surgical procedure performed, consent withdrawn shortly before the operation). As randomisation takes place up to 72 hours before the operation for organisational reasons, the patient is included in the clinical trial from this point in time. Without the surgical procedure or without the use of a biomesh, there is no data to be collected. Patients who are excluded from the trial after randomisation and before the start of the observation period are replaced by another trial participant in order to achieve the required number of cases. The randomisation sequence is continued, i.e. the following patient does not receive the randomised allocation of the patient who dropped out. The study will continue until the required number of analysable patients has been reached.

4.4.1. Procedure in the event of premature termination of treatment in the clinical trial

For all study participants who terminate the study prematurely (drop outs), the reasons for dropping out should be documented if possible. According to the IEHS guidelines, recurrences after TEP or TAPP are treated using the Lichtenstein approach (anterior approach). The

occurrence of a recurrent hernia on one side does not lead to premature termination of the study for this patient, as observation of the hernia on the other side must be continued. Only when a recurrence requiring surgery has also occurred on the other side the study ends for this patient.

4.5. Closure of trial centres/early termination of the clinical trial

The sponsor is authorised to terminate the trial prematurely due to relevant medical or ethical concerns or lack of feasibility of the trial. In such a case, the reasons for the premature termination of the trial will be documented in detail. Patients who are still undergoing treatment at the time the trial is terminated will continue to be monitored as planned with regard to the occurrence of recurrence (follow-up). If an investigator has ethical concerns regarding the continuation of the trial, this must be reported immediately to the LKP.

Early termination of the clinical trial will be if

- the risk-benefit ratio for patients has changed significantly,
- the use of one of the surgical procedures is no longer justifiable,
- the sponsor deems it necessary to discontinue the clinical trial for safety reasons (e.g. on the advice of the DSMC),
- the clinical trial proves to be unfeasible.

The sponsor decides on the termination of the study in agreement with the LKP.

4.6. Treatments

4.6.1. Treatments used

Patients with bilateral inguinal hernias will undergo an operation that corresponds to routine laparoscopic hernia surgery (TAPP or TEP) and is performed according to the guidelines of the IEHS (International Endohernia Society) after consenting to participate in the study. The only deviation is that one side is specifically treated with one side is supplied with a synthetic mesh material and the other with biological mesh material. Randomisation is used to decide which side is supplied with the biological mesh material. The other side is then supplied with the synthetic counterpart. Both sides must be treated using the same technique (TAPP or TEP). The implant remains permanently in the body or, in the case of the biological mesh material, is replaced by the body's own tissue; follow-up treatment is not normally necessary.

4.6.2. Description of the medical device

The aim of this study is not to specifically investigate the effectiveness of a single manufacturer's mesh, but to show whether there is a general advantage for biological mesh material over synthetic mesh material in everyday use. Meshes from different manufacturers are available for both study arms. Only CE-certified meshes will be authorised. The synthetic mesh materials should be made of polypropylene, polyester or PVDF, have large pores and be lightweight (< 100 g/m$^{2)}$. The biological mesh materials should consist of a perforated, non-crosslinked, acellular and collagenous matrix. The mesh sizes used should be at least 10x15 cm. The meshes can be fixed with adhesive.

In addition, the participating centres should use the meshes that is also used in their clinical routine or that they would order under the conditions, on the condition that this merchandise has a valid CE certificate for the required purpose.

Form of applikation: Laparoscopic implantation according to IEHS guidelines

4.6.2.1. Labelling of the medical device

As commercial goods are used, no separate labelling is required..

4.6.2.2. Storage of the medical device

The storage conditions are to be taken from the application information of the commercial product used by each centre.

4.6.3. Method for assigning patients to the treatment groups and blinding

All patients included in the study are assigned a unique patient identifier. This consists of the abbreviation of the trial centre and the consecutive number of the included patient. As all included patients have a bilateral hernia, the randomisation only has to determine which side is operated on and how. The patient is therefore represented in both study groups, with one hernia in each study arm. Randomisation is therefore only between the following two options: "Right side biological and left side synthetic mesh" and "Left side biological and right side synthetic mesh".

Before the start of the study, the biometrician creates a randomisation list for each centre, which assigns the mesh material to the respective treatment side (right/left). Randomisation is stratified by centre and performed as block randomisation (block size 6-10). The randomisation results are announced centrally by telephone by the secretariat of the LKP in Cologne-Merheim (0221/8907-3770) during regular working hours (elective procedure) in order to monitor the

progress of the study permanently and to be able to unblind the patient in the event of a recurrence (see below). The person entrusted with this task is not involved in the further steps of the study (e.g. follow-up).

As the surgical technique is identical for both sides, it is not possible to be unblinded from the outside by different scars or dressings postoperatively. Both the patient and the hospital staff (except the surgeon) are therefore blinded.

The surgeon cannot necessarily be blinded. Therefore, the follow-up should not be carried out by the surgeon (possibly by someone else from the surgical team).

In the event of a hernia recurrence that necessitates a reoperation, the affected side is unblinded by telephone by the LKP secretariat to enable adequate preparation for the operation. This automatically unblinds the unaffected side as well. When a hernia occurs, the study endpoint is reached for this hernia, but not for the patient. As long as the other side has not yet developed a recurrence, the patient remains in the study with the other hernia. Only when the other hernia also develops a recurrence is this patient's participation in the study terminated.

4.6.4. Previous therapy and concomitant therapy

Given the existing indication and elective surgery, this point is irrelevant for the study. Any contraindications are listed in the exclusion criteria.

4.6.4.1. Alternative therapy in an emergency

As these are elective procedures, no emergencies requiring special alternative therapies are to be expected. Any recurrences are treated as described under 4.4.1.

4.6.5. Further treatment after the end of the clinical trial

In principle, the patient is already recovered after discharge and no further treatment is necessary (see also 4.6.1).

4.7. Efficacy and safety parameters

4.7.1. Measurement of efficacy and safety parameters

4.7.1.1. Primary and secondary outcomes

Pain reduction is measured postoperatively and during follow-up examinations using the 0-10 point visual analogue scale (VAS). Each side of the operation is documented separately. Pain

recording is also limited to the groin area so as not to impair data quality due to other sources of pain (headache and back pain, for example). As with pain recording, the recurrence rate is also recorded separately for each side. In the event of a recurrence, the result of a clinical examination must be confirmed diagnostically (ultrasound/CT/MRI). In the absence of follow-up examinations, the LOCF principle (Last Observation Carried Forward) is applied. The recurrence rate requires at least the data from the 6-month follow-up.

4.7.1.2. Further outcomes

In addition to pain and recurrence rates, complications and patient satisfaction are also recorded. For this purpose, the type and number of complications (see also 4.9.1 and 2.3) are analysed, separately for each side where appropriate. The evaluation is performed using the McNemar test for the event rate in paired data.

For patient satisfaction, a side-by-side, descriptive evaluation of the assessment of the overall result, the foreign body sensation and the occurrence of paraesthesia is carried out.

4.7.1.3. Safety analysis

After discharge and after the follow-up visits of a patient, the study centre must inform the LKP/study management promptly (within 7 days) of any complications that have occurred. In the event of a conspicuous accumulation of complications, the LKP together with the DSMC will assess whether these are (serious) adverse effects of the products (ADE or SADE) and whether the conduct of the study should be changed or the study terminated prematurely if necessary

4.7.1.4. Description of the individual visits

Table 2 Flow Chart

			**Behandlung**		
	**Screening**	**Preoperative preparation**[3]	**Surgery**<br><br>**D1**	**Discharge**[3]	**Follow-Up**<br>**1 Week +/- 72h**<br>**6 Months +/- 4 Weeks**<br>**12 Months +/- 2 Months**<br>**24 Months +/- 2 Months**<br>**5 Years +/- 2 Months**<br>**10 Years +/- 2 Months**
Patient identification	X				
Information		X			
Informed Consent		X			
Pain: VAS (0-10), side-separated	X	X		X	X
Randomisation		X			

Surgery			X		
Imaging (Sonography, MRI, CT)	(X)[2]	(X)[2]		(X)[2]	(X)[1]
Complications			X	X	X
Recurrence				X	X
Wound infections				X	X
Haematoma/Seroma				X	X
Patient satisfaction					X

[1]In case of suspected recurrence
[2] These measures are not mandatory for the study and are carried out if requested by the surgeon or anaesthetist. The necessity results from the patient's risk profile.
[3] No time limits are for preoperative preparation and discharge.

Screening-Visit

The screening visit takes place as part of the clinical routine, during which a patient routinely presents in the consultation, the emergency outpatient department or as part of a consultation. If the patient fulfils the inclusion criteria (all examinations that lead to a diagnosis correspond to the clinical routine), initial information about the study and the planned surgical procedure (TEP or TAPP) is provided. The patient receives an appointment for surgery regardless of participation in the study. In addition, an initial side-separated pain assessment is carried out using VAS. If necessary, an imaging procedure (sonography/CT/MRI) is also performed to support the diagnosis. However, this is independent of participation in the study and is part of routine clinical practice.

Preoperative preparation (D-3 bis D-1)

The patient comes to the clinic for routine surgery preparation (3-1 days before the operation). A written declaration of consent for the operation and anaesthesia is mandatory. Optional laboratory tests (e.g. small blood count, electrolytes, Quick/INR/PTT, creatinine, CRP, TSH) can be carried out if requested by the surgeon or anaesthetist (the sole use of a biomesh does not require a blood sample, the necessity is determined by the patient's risk profile).

However, the patient is also informed about the study by the investigator or a medical member of the study group and asked for consent to participate in the study.

If consent is given, a randomisation list drawn up in advance is used to allocate which side will be supplied with the biological mesh by telephoning the LKP secretariat. The other side then automatically receives the synthetic mesh.

Surgery (D1)

The operation must be standardised in accordance with the "Guidelines for laparoscopic (TAPP) and endoscopic (TEP) treatment of inguinal hernia" published by the International Endohernia Society (IEHS). This is ensured by the fact that only hernia centres participate in the study.

Both sides must be treated with the same surgical procedure (TEP or TAPP), the only difference being the mesh used (biomesh or synthetic mesh).

Complications are recorded for the first time (see also 2.3 and 4.9.1.3)

Hospital discharge

On the day of discharge, pain is again recorded using VAS and any complications are documented. If the patient's profile makes it necessary, additional imaging procedures are carried out. This is not mandatory for study reasons, but will be documented if available.

The use of a biomesh is not expected to prolong hospitalisation.

Follow-up (1 week +/- 72h, 6 months +/- 4 weeks, 12 months +/- 2 months, 24 months +/- 2 months, 5 und 10 years +/- 2 months postoperative)

A clinical examination is performed and pain is assessed separately on each side using the VAS (visual analogue scale 0-10). In addition, the presence of a recurrence is evaluated. In the event of a suspected clinical recurrence, confirmation should be made by means of sonography, MRI or CT, which also corresponds to clinical routine.

The incidence of pain after 6 months and the occurrence of a recurrence within the first two years are the two primary endpoints.

Patients can optionally consent to further follow-up visits five and ten years after the operation. The examinations correspond to those of the earlier follow-up visits. If the patients no longer live at the location of the trial centre after ten years, they can be contacted by telephone and asked about the occurrence of recurrences.

The following are also collected

- Complications (e.g. revision surgery, mesh dislocation, haemorrhage)
- Wound infections
- Haematoma/ Seroma

- Patient satisfaction (overall result, foreign body sensation, paraesthesia)

Duration of the clinical trial in the individual patient

The inpatient stay for the patient is usually completed after 1-6 days; due to the necessary follow- up examinations, the total duration of the study for each patient is approx. 2 years. At the follow-up examination two years after the operation, the patient can decide whether they would like to take part in further check-ups, which take place five and ten years after the operation. If the patient agrees to further participation in the study, the entire study lasts for the patient until a recurrence occurs on both sides, but for a maximum of ten years.

4.7.2. Appropriateness of the measurement methods

The primary objective is to investigate whether a significant reduction in pain intensity can be demonstrated when a biological mesh is used instead of a synthetic mesh for the treatment of inguinal hernias. This requires a rational assessment of subjective pain intensity, as can be achieved with the commonly used visual analogue scale (VAS). At the same time, it should be shown that the biological mesh is not inferior in terms of recurrence and/or procedure-related complications. To this end, it is necessary to determine the recurrence rate and to record the type and number of complications that occur.

The self-controlling design of this study allows an ideal comparison between the two mesh materials. As only patients with bilateral hernias are included and one of the two meshes is inserted on each side (using the same surgical technique, the patient is blinded), each patient is simultaneously their own control. This enables a comparison that is almost completely unaffected by other influences such as comorbidity, age, gender or experience of the surgeon.

4.8. Ensuring data quality

4.8.1. Monitoring

Monitoring is carried out at the trial centres to ensure the quality of the trial. The aim of monitoring is to verify the safeguarding and protection of the rights and safety of trial participants, the validity, verifiability and completeness of the trial data and the compliance of the trial conduct with the trial protocol, GCP and the applicable legal provisions.

All investigators agree that the monitor shall visit the study site at regular intervals and that the study sites shall provide the investigator with the necessary information. This is agreed in the

study site contracts. A corresponding passage is included in the declaration of consent (see Section 5.4), which grants the monitor the right to compare the documentation forms (CRF) with the original documents (medical records, ECG, laboratory printouts, etc.), taking into account the Data Protection Act. The auditors allow the monitor direct access to all necessary documents for the purposes of audit-related monitoring. The purpose of these visits is in particular:

- checking the informed constent forms,
- Patient safety checks (occurrence and documentation of complications)
- checking the CRFs for accuracy and completeness,
- the validation of CRFs against the original data (Source Data Verification, SDV)
- the evaluation of the progress of the examination,
- Checking compliance with the study protocol,
- GCP-compliant conduct of the study at the study site
- Discussion with the investigator about the conduct of the study and any deficiencies identified

A monitor report is prepared for each visit, which documents the progress of the clinical trial and provides information on any difficulties encountered (e.g. refusal of access).

The exact scope and type of monitoring are described in a separate monitoring manual..

4.8.2. Audits/Inspections

The sponsor has the right to conduct audits at the study sites and other facilities involved in the trial as part of quality assurance. The aim of audits is to check the validity, verifiability and completeness of the data and the credibility of the clinical trial as well as to check the safeguarding of patient rights and the guarantee of patient safety. The sponsor may commission persons who are not otherwise involved in the clinical trial (auditors) for this purpose. These persons are permitted to inspect all trial-related documents (in particular: Study protocol, data collection forms, patient files, documentation of study medication, study-related correspondence).

The sponsor and all participating study sites undertake to support auditors, inspections by competent authorities and, in this context, to grant the authorised persons access to the original documents.

All persons carrying out audits undertake to treat personal data and other data confidentially.

4.9. Documentation

All study-relevant data is promptly recorded by the responsible investigator in the documentation forms (CRF) provided. The documentation can be delegated to other members of the study team. The survey forms are signed personally by the investigator. The information on patient satisfaction is only documented in the CRF, so that this part of the CRF serves as the source document. All other data recorded in the CRF is taken from the patient file.

4.9.1. Data management

In this study, the primary data collection is carried out using paper-based documentation forms. The data collected will be entered into a database at the IFOM. The data is entered using a data management programme that meets the FDA requirements according to 21 CRF part 11. The programme has an audit trail for tracking the entries and a role allocation for the users (data manager, data entry, monitors, etc.). The software is integrated into an IT infrastructure with an appropriate security concept and backup system. The electronic database is set up by the IFOM's data management team. The study-specific system, i.e. the documentation forms and the data collected with them, are validated. In addition, check processes are defined for the input (edit or validation checks in the front end or back end). After the validation process, the data is finally entered into a production version. This means that an electronic check sheet set that can only be used at IFOM is used for data entry. Details on the implementation of the data management system, the validation and checking processes are defined in the data management SOPs and in the data management plan.

The first step in processing the data in data management is to register and secure the incoming paper-based test forms. For this purpose, a copy of the test forms is stored in electronic form on an external server. The data is then entered by trained data entry staff. During data entry, the data is checked again by the data entry staff or the data management programme's checking system. In the event of queries, missing values or discrepancies, specific queries are sent to the centre. A separate query process is started for this purpose. The queries are registered and sent to the study sites. The responses confirmed by the investigator are entered into the database. In addition to the data providers, the monitors also use this query process for queries that cannot be clarified directly at the centre. Furthermore, there are corrections that can be made independently by the data entry person. The circumstances under which these can be carried out are specified in writing. The term "self-event corrections" refers to corrections that affect free text, for example, or that no longer require a query process in an

unambiguous situation. Example: standardised input of free text with regard to upper and lower case, if the examining physicians have only used capital letters, input of free text umlauts as ü=ue, ö=oe, ä=ae etc., in order to problems when converting the texts into other database formats. Standardised information if e.g. units are used, e.g. "mg/l" and not "mg / l" or similar. If a field is filled in but the corresponding yes/no query has not been specified, i.e. if it is clear that the answer can clearly only be "yes" or "no" (this procedure was suggested by an auditor in other studies). All so-called "self event corrections" are set out in writing in a list or a "Note To File" (see second case). As rule, this list is created during the test entry of study data. The list is signed off by the project and data manager. The data entry persons are trained in this regard. The data entry persons may only enter data within the scope of these defined "corrections". It is not possible to correct the content.

4.9.1.1. CRF: Demographic data and preoperative data

(Screening/inclusion, collection in consultation or outpatient clinic or consultation, missing data can be added during preoperative preparation (e.g. medication))

- Demographic data
  - Date of birth
  - Gender
  - Size
  - Weight
  - BMI
- Inclusion criteria:
  - Primary, bilateral inguinal hernias (if necessary, imaging confirmation by sonography/CT/MRI), if necessary, indication if one hernia is larger than the other (should "randomise out" in the end anyway)
  - Patient suitable for laparoscopic hernia surgery
  - Age >18
- Exclusion criteria:
  - Recurrent hernia
  - Incarcerated hernia
  - Acute systemic infection
  - Expected non-compliance with any study requirement
  - Severe comorbidity (ASA >/=4)
  - Life expectancy <12 months
  - Chemotherapy within the last 4 weeks

- Radiotherapy within the last 2 months
    - Known allergy to pig or bovine antigens
    - Simultaneous participation in another study that interferes with this study
    - Previous participation in this study
- Pain according to VAS (0-10) separated by side
    - At rest
    - Under strain (coughing/pressing)
- Previous abdominal operations (e.g. appendectomy, cholecystectomy, etc.) with year of previous operation
- Concomitant diseases (e.g. CHD, diabetes mellitus, tumours, etc.)
- Concomitant medication incl. medication on demand
- ASA-classification

4.9.1.2. Preoperative preparation (D-3 bis D-1)

- Pain according to VAS (0-10) separated by side
    - At rest
    - Under strain (coughing/pressing)
- Information/consent

4.9.1.3. CRF: Operation data

- Randomisation
- Patient treated as randomised?
- Date
- Duration of surgery (incision-suture)
- ASA classification
- Antibiotic prophylaxis yes/no?
- Adhesiolysis necessary yes /no
- Type of hernia (per side)
    - Medial/ lateral/ combined (classification according to Schumpelick)
    - Size (1=<1.5 cm, 2=1.5-3 cm, 3=>3 cm, classification according to Schumpelick)
- Drainage yes/no
- Fixation of the mesh yes/no, which adhesive
- Complications
    - Intestinal injury

- Bleeding incl. severity (mild/ moderate/ severe)
- Injury to the bladder
- Conversion
- Further interventions:
  - Removal of Meckel's diverticulum
  - Simultaneous umbilical hernia operation
  - other

4.9.1.4. CRF: hospital course/discharge (D2-D4)

- Number of inpatient treatment days
- Drainage removal when (if drainage inserted)
- Wound healing of the surgical accesses regular?
- Pain according to VAS (0-10) separated by side
  - At rest
  - Under strain (coughing/pressing)
- If sonography is performed (optional, not mandatory)
  - Seroma/haematoma laterally separated
  - mesh dislocation laterally separated
- Complications
  - Infection (local/systemic)
  - Seroma/Haematoma
  - Thromboembolism
  - Ileus
  - Revision operation separated laterally
  - mesh dislocation laterally separated
  - other
- Patient satisfaction
- Feeling of foreign body yes/no separated from the side
- Paraesthesia yes/no separated by side

4.9.1.5. CRF: Follow-up 2 years by centres with consultations:

1 week postoperative (+/-72h), 6 months postoperative (+/- 4 weeks), 1 year postoperative (+/- 2 months), 2 years postoperative (+/- 2 months)

- Date of the follow-up
- Re-hospitalisation (number and duration, date of 1st re-hospitalisation)
- Wound healing of the surgical accesses regular?

- Pain according to VAS (0-10) separated by side
    - At rest
    - Under strain (coughing/pressing)
- Side-separated recurrence
    - In the event of a suspected recurrence, imaging must be performed (sonography/CT or MRI)
- If sonography or other imaging is performed (optional, not mandatory)
    - Seroma/haematoma
- Complications
    - Intestinal injury
    - Bleeding incl. severity (mild/ moderate/ severe)
    - Injury to the bladder
    - Conversion
- Patient satisfaction
- Feeling of foreign body yes/no separated from the side
- Paraesthesia yes/no separated by side

4.9.2. Archiving

All documentation forms, declarations of consent and other important trial documents are stored for at least 15 years in accordance with Section 12 (2) MPG. The patient identification list is kept separately from the trial documentation.

5. Ethical and regulatory aspects

5.1. Independent ethics committees

The clinical trial is only started after a favorable assessment by the responsible ethics committee has been received. In each additional trial centre, the clinical trial is only conducted after the responsible ethics committee involved has determined the suitability of the trial site and the qualification of the investigators.

5.2. Ethical conduct of the clinical trial

This protocol and any subsequent amendments to the protocol have been or will be drawn up in accordance with the current version of the Declaration of Helsinki.

5.2.1. Legal provisions, guidelines and standards taken into account

The present clinical trial is conducted in accordance with the published principles of Good Clinical Practice for medical devices (EN ISO 14155-1 and -2; 2003) guideline and the applicable legal requirements (in particular the Medical Devices Act and the Ordinance on Clinical Trials of Medical Devices), due to the study design (CE-certified medical devices in indication §23b MPG) but to an adapted extent. These principles concern, among other things, ethics committee procedures, patient information and informed consent, compliance with the protocol, administrative documents, documentation of trial medication, data collection, patient records (source documents), recording and reporting of complications, preparation of inspections and audits and retention of documents. All investigators and other personnel directly involved in the trial are informed that domestic and foreign regulatory authorities, the competent federal authorities and personnel authorised by the sponsor of the clinical trial are entitled to inspect study documents and patient files at any time.

5.3. Notifications to authorities, authorisations and registration

Since the present study is a study with CE-certified medical devices in the intended indication and no additional invasive measures are necessary for the study, no authorisation is required according to §23b MPG (German Medical Devices Act).

The clinical trial is also registered with a trial registry recognised by the WHO (e.g. www.clinicaltrials.gov / DRKS).

5.4. Information and consent of the test subjects

A patient can only be included in the clinical trial if he/she has given his/her consent after having been informed verbally and in writing by an investigator about the nature, significance and scope of the clinical trial in an appropriate and comprehensible manner. At the same time as giving consent, the patient must have declared that he/she agrees to the recording of data in the course of the clinical trial and its review by persons authorised by the sponsor (e.g. monitor, auditor) and by the competent supervisory or federal authority. The patient is informed about the potential benefits and adverse effects of the investigational product and about the necessity and significance of a clinical trial. It must be clear to them that they can withdraw their consent at any time and without giving reasons, without suffering any disadvantages as a result.

The original of the written informed consent will be kept in the study folder of the trial centre. The patient will be given a copy of the written patient information including a copy of the insurance confirmation and conditions as well as the declaration of consent. In addition, a copy of both documents will be filed in the patient's file.

Patient information and declaration of consent are attached.

Patient information and consent forms as well as all other documents that participants receive are submitted to the responsible ethics committee for approval before use. As part of the monitoring process, it is checked whether the current consent form was personally dated and signed by the patient concerned before the start of the clinical trial..

5.5. Clinical trials insurance

As this is a study with CE-certified medical devices in the indication and therefore any consequential damage is covered by the product liability or public liability of the participating centres, separate clinical trials insurance is not necessary in accordance with Section 23b MPG.

Travel accident insurance is taken out for the study-related additional follow-up visits. Details of the insurance will be given in the patient information leaflet, or a copy of the insurance policy will be provided.

5.6. Data protection

The provisions of the data protection laws are observed. It is ensured that all research materials and data are adequately pseudonymised in accordance with data protection regulations prior to scientific use.

The trial participants are informed about the transfer of their pseudonymised data to the recipients named therein as part of the documentation and notification obligations in accordance with point 4.74 of Good Clinical Practice for Trials with Medical Devices (EN ISO 14155-1 and -2; 2003). Persons who do not consent to the disclosure will not be included in the clinical trial.

6. Statistical methods and determination of the number of cases

6.1. Statistical and analytical plan

A statistical analysis plan is drawn up for the study before patient recruitment begins.

The unit of investigation in this study is the hernia, not the patient. Due to the self-controlled design of the study, both study arms are always the same size.

At least one follow-up examination is required to determine the main endpoint "recurrence". Missing follow-up examinations are replaced using LOCF (Last Observation Carried Forward), whereby this principle is also applied backwards, i.e. if a 12-month result is available but not at 6 months, the 12-month data is also used "backwards" for the 6-month results. If no single follow-up examination is available, missing values are not replaced; such cases are not included in the analysis of the "recurrence" endpoint.

6.1.1. Study populations

The study is evaluated according to the intention-to-treat (ITT) principle, i.e. the right or left hernia is allocated to the group with biological mesh or synthetic mesh according to randomisation.

If mix-ups occur during the course of the study, i.e. the wrong sides were operated on (i.e. right/left instead of left/right), or both sides were operated on using the same procedure, then a protocol violation has occurred. In this case, a "Per-Protocol" (PP) analysis and an "As Treated" analysis are also carried out. In the per-protocol analysis, patients with protocol violations are excluded; in the as-treated analysis, the respective hernia is assigned to the material group (biological or synthetic).

Surgery-specific complications are also reported according to "As-Treated".

6.1.2. Description of the patient collective

The patient population is identical in both study arms (biological / synthetic), as it is a self-controlled design. Therefore, the included patients are only described descriptively. For the hernias, however, specific details (size; side; pain) are recorded separately for both sides and compared according to the allocation.

6.1.3. Primary outcomes

In this study, two primary endpoints are considered in parallel: pain intensity and recurrence. The total error of the first type (α=0.05) is distributed equally between the two endpoints according to a Bonferroni adjustment (α=0.025 in each case).

Pain intensity is recorded at 6 months after the operation using a visual analogue scale (VAS; 0-10 points) and tested for difference. Two pain values are recorded for each patient, separately for the left and right groin area. This means that dependent data is available. As experience shows that pain values are not symmetrically distributed, the evaluation is carried out non-parametrically with a rank test (Wilcoxon's rank sum test). The p-value is 0.025.

The second primary endpoint is the recurrence rate. The occurrence of a recurrence is recorded at several follow-up examinations over a period of 2 years after the procedure. A recurrence must confirmed diagnostically. This endpoint will be tested for non-inferiority. It is expected that the recurrence rate with a biological mesh will be lower, the same or at most 3 % higher than the recurrence rate with a synthetic mesh. A range of +/- 3 % is thus defined as still comparable. A 95 % confidence interval is determined for the observed difference in recurrence rates (biological - synthetic). If the value of +3 % is not within this confidence interval, the biological mesh is considered non-inferior. Non-inferiority is a one-sided question. The 95 % confidence interval here corresponds to a one-sided question with a p-value of 2.5 %, as required by the Bonferroni correction.

6.1.4. Secondary outcomes

The secondary outcomes are presented descriptively and compared exploratively. The recurrences are visualised using Kaplan-Meier curves.

Complications are compared in the as-treated collectives.

6.1.5. Determination of the number of cases

First endpoint: Pain: Measurements of pain intensity using VAS are known to show high variability. Postoperative values show standard deviations (SD) of 1.5 - 2.0 points, while in follow-up examinations the mean values as well as the dispersion tend to be lower (SD 1.0 - 1.5). A larger proportion of patients will also have no pain at follow-up. In the present study, we expect this to be the case for about half of the hernias for both materials. Patients with pain mostly report intensities <5 points. An expected difference of one point is therefore considered too large. A difference of 0.5 points is therefore planned here. With an SD of 1.5 points, this would mean a reduction of one third of the standard deviation. The required number of cases

to detect such a difference (paired t-test; power 90 %; α=0.025) would be 114 hernias per group, i.e. 114 patients. As a non-parametric rank test is planned for the analysis, an additional 15% is added to the number of cases, i.e. 131 hernias per group, or 131 patients.

The second endpoint "recurrence" is a non-inferiority hypothesis. A recurrence rate of approx. 5 % is expected over a period of two years (synthetic meshes). The maximum acceptable difference to the recurrence rate for the biological meshes is +3 %, i.e. a maximum recurrence rate of 8 %. If both meshes have the same recurrence rate (e.g. 5 %), then 203 cases are required to rule out a difference of more than 3 %, i.e. the 95 % confidence interval around 0 % is then [-3; +3]. If the recurrence rate for the biological meshes is 1 % higher than for the synthetic meshes, then 451 cases would be required for a 95 % confidence interval of +/- 2 %, i.e. the confidence interval around the difference of +1 % would then be [-1; +3]. This means that a difference > 3 would also be outside the confidence interval. As this is a one-sided question, a difference > 3 % can only be achieved with a p<0.025.

Thus, the second endpoint (recurrence) requires a larger number of cases (n=451). If one also assumes a follow-up loss of approx. 10 %, this figure increases to a total of 496 people (with bilateral hernias).

The number of patients to be screened is 10% higher: n=546.

7. Use of the data and publication

7.1. Reports

7.1.1. Interim reports

No interim reports are planned.

7.1.2. Final report

The ethics committee will be informed of the termination of the clinical trial within 90 days.

Within one year of completion of the clinical trial, the summary of the final report on the clinical trial covering all significant events of the trial is submitted to the responsible ethics committee.

7.2. Publication

It is planned to present the results of the clinical trial in a scientific journal and/or at German and international congresses in due course and after mutual agreement with the LKP. In principle, preference should be given to publication of the clinical trial as a whole. The "Uniform requirements for manuscripts submitted to biomedical journals (International Committee of Medical Journal Editors" (ICMJE) [JAMA 1997;277:927-34]) are taken into account.

Registration of the clinical trial in a public register in accordance with the recommendations of the ICMJE is also planned (see also 5.3).

For all publications, data protection is maintained for all data of study participants as well as for the data of participating investigators. The success rates and individual results of the participating trial centres are only known to the sponsor.

Publication or presentation of the results of this clinical trial, including publication or presentation by a single trial site, is subject to prior acknowledgement and prior comment and approval by the sponsor.

By signing the declaration of participation, the investigator agrees that the results of this clinical trial may be submitted to national and international regulatory and supervisory authorities, the German Medical Association, the National Association of Statutory Health Insurance Physicians and the health insurance funds. At the same time, the investigator agrees that his/her name, address, qualifications and the extent of his/her participation in the clinical trial may be disclosed in this context.

In the case of publications, support from the ZKS-UW/H is indicated. Where applicable, contributors to ZKS-UW/H projects are listed as co-authors. If the contributors are not co-authors of the article, they are named in the acknowledgements. A copy of all project publications must be made available to the ZKS-UW/H.

8. Changes to the study protocol

In order to ensure largely comparable conditions in all trial sites and in the interests of proper data evaluation, no changes are planned to the agreed study conditions laid down in the study protocol. In exceptional cases, however, changes to the trial conditions are possible. These will only be made after mutual agreement between the sponsor, the sponsor representative, the LKP and the biometrician as well as all signatories (authors) of this protocol.

This does not apply to changes to the protocol that must be made directly for the safety of the patient.

Any change to the study procedures provided for in the protocol must be made in writing, stating the reasons for the change, and must be signed by all authors of the protocol (amendment).

Subsequent changes are submitted to the Ethics Committee for review and, if they concern changes that affect patient safety, data quality and/or data protection, are only implemented after the Ethics Committee has issued an opinion. This does not apply to changes that are necessary to avert immediate danger.

9. Literature

1. Rutkow IM. Epidemiologic, economic and sociologic aspects of hernia surgery in the United States in the 1990s. Surg Clin North Am. 1998. 78:941-951.
2. Zendejas B, Ramirez T, Jones T, et al. Incidence of inguinal hernia repairs in Olmsted County, MN: a population-based study. Ann Surg. 2013. 257:520-526.
3. Primatesta P, Goldacre MJ. Inguinal hernia repair: incidence of elective and emergency surgery, readmission and mortality. Int J Epidemiol. 1996. 25:835-839.
4. Reaveley AM, Nguyen-Van-Tam JS, Logan RF. Who dies from hernia? J Epidemiol Community Health. 1998. 52:532-3
5. Charlton JRH, Hartley RM, Silver R, et al. Geographical variation in mortality from conditions amenable to medical intervention in England and Wales. Lancet. 1983. i:691–6.
6. Bay-Nielsen M, Kehlet H, Strand L, et al. Quality Assessment of 26,304 Herniorrhaphies in Denmark: a Prospective Nationwide Study. Lancet. 2001. 358:1124-1128.
7. Murray CJL, Vos T, Lozano R, et al. Disability-adjusted Life Years (DALYs) for 291 Diseases and Injuries in 21 Regions, 1990–2010: A Systematic Analysis for the Global Burden of Disease Study 2010. Lancet. 2012. 380:2197-2223.
8. Macintyre IMC. Best practice in groin hernia repair. Br J Surg. 2003. 90:131–132.
9. Karthikesalingam A, Markar SR, Holt PJ, et al. Review Meta-analysis of randomized controlled trials comparing laparoscopic with open mesh repair of recurrent inguinal hernia. Br J Surg. 2010. 97:4-11.
10. Haapaniemi S, Gunnarsson U, Nordin P, et al. Reoperation after recurrent groin hernia repair. Ann Surg. 2001. 234:122–126.
11. Nienhuijs S, Staal E, Strobbe L, et al. Chronic pain after mesh repair of inguinal hernia: a systematic review. Am J Surg. 2007. 194:394–400.
12. Poobalan AS, Bruce J, Smith WC, et al. A review of chronic pain after inguinal herniorrhaphy. Clin J Pain. 2003. 19:48–54.
13. Bochicchio GV, Jain A, McGonigal K. Biologic vs Synthetic Inguinal Hernia Repair: 1-Year Results of a Randomized Double-Blinded Trial. J Am Coll Surg. 2014. 218:751-7.

14. Agresta F, Bedin N.Transabdominal laparoscopic inguinal hernia repair: is there a place for biological mesh? Hernia. 2008. 12:609-12.
15. Köckerling F, Alam NN, Narang S et al. Biological Meshes for Inguinal Hernia Repair – Review of the Literature. Front Surg. 2015. 2: 48.
16. The European Agency for the Evaluation of Medicinal Product. Note for Guidance on Good Clinical Practice (CPMP/ICH/135/95).
17. Sevonius D, Gunnarsson U, Nordin P et al. Recurrent groin hernia surgery. Br J Surg. 2011 Oct;98(10)
18. The European Agency for the Evaluation of Medicinal Product. Note for Guidance Structure and Content of Clinical Study Reports (CPMP/ICH/137/95).
19. National Cancer Institute. Protocol Templates, Applications and Guidelines http://ctep.cancer.gov/guidelines/templates.html.
20. EMEA-Guideline On Data Monitoring Committees: EMEA/CHMP/EWP/5872/03 Corr
21. The DAMOCLES Study Group. A proposed charter for clinical trial 2005 data monitoring committees: helping them do their job well. Lancet 2005; 365: 711-22
22. Clinical trial registration: a statement from the International Committee of Medical Journal Editors. Accessed at http://www.icmje.org/clin_trial.pdf on 22 May 2007.
23. WHO. Causality Assessment of Suspected Adverse Reactions. http://www.who-umc.org/DynPage.aspx?id=22682

10. Attachments

10.1. Participating trial sites and principal investigators

No	Centre	Prinicpal investigator
01	Kliniken der Stadt Köln, Klinikum Köln Merheim	Prof. Dr. Markus M. Heiss
02	Asklepios Westklinikum Hamburg	Prof. Dr. Dr. Thomas Carus
03	Hernienzentrum PAN-Klinik Köln	Dr. Bernd Stechemesser
04	St. Elisabeth-Krankenhaus Dorsten	PD Dr. Wilhelm Gross-Weege
05	GFO Kliniken Rhein-Berg.	Dr. Wolfgang Spangenberger
06	Wilhelmsburger Krankenhaus Groß-Sand	Dr. Wolfgang Reinpold
07	St. Marien-Krankenhaus Ahaus-Vreden	Dr. Moritz Meyer
08	Helios Klinik Attendorn	Dr. Klaus Friedhoff
09	Johanniter-Hospital Bonn	Dr. Nicola Cerasani
10	Josephs-Hospital Warendorf	Prof. Dr. Christoph Seiler
11	Klinikum Leverkusen	Dr. Dirk Antoine
12	DRK-Krankenhaus Luckenwalde	Dr. Jan Dornbusch
13	St. Barbara-Klinik Hamm-Heessen	Dr. Dietmar Picke
14	GRN-Klinik Weinheim	Dr. Frauke Hildebrandt
15	Ev. Diakonissenhaus Leipzig	Dr. Niels-Torsten Hoedt
16	Eduardus-Krankenhaus Köln	Dr. Jörg Weber
17	Lukaskrankenhaus Neuss	Dr. Bernhard Lammers
18	Uniklinik Aachen	Dr. Mark Schneider
19	Ev. Krankenhaus Köln Weyertal	PD Dr. Claudia Rudroff
20	St. Bernhard-Hospital Kamp-Lintfort	Prof. Dr. Gernot Kaiser
21	Ammerland Klinik Westerstede	Dr. Muneer Deeb
22	Dreifaltigkeits-Krankenhaus Wesseling	Prof. Dr. Christoph Jacobi

10.2. Patient information and declaration of consent
