# Supplementary material for: Biological vs Synthetic Mesh in Laparoendoscopic Inguinal Hernia Repair: The BIOLAP Randomized Clinical Trial
Source: JAMA Surg. 2025 Oct 8;160(12):1309–16. doi: 10.1001/jamasurg.2025.4071 (PMC12509081; doi:10.1001/jamasurg.2025.4071)
Supplement: Supplement 2. — Statistical Analysis Plan [file jamasurg-e254071-s002.pdf]

Statistical Analysis Plan (SAP)

BIOLAP Study

Biological versus synthetic mesh in laparoscopic hernia repair - a randomised multicentre, prospective, self-controlled clinical trial

Status: 30 July 2019

Statistician responsible:

Prof Dr Rolf Lefering

Institute for Research in Operative Medicine

Witten/Herdecke University

Ostmerheimer Straße 200, House 38

51109 Cologne, Germany

1. Synopsis of the study

Sponsor:	Witten/Herdecke University<br>Alfred-Herrhausen-Straße 50, 58448 Witten, Germany<br>represented by:<br>Centre for Clinical Trials at Witten/Herdecke University Alfred-Herrhausen-Straße 50, 58448 Witten, Germany
Head of the clinical trial:	Prof Dr med M. Heiss<br>Visceral, Vascular and Transplant Surgery, Cologne Merheim Medical Center, Ostmerheimer Str. 200, 51109 Cologne, Germany<br>+49(0) 221 89073770, HeissM@kliniken-koeln.de
Title of the clinical trial:	BIOLAP: Biological versus synthetic mesh in laparoscopic hernia repair - a randomised multicentre, prospective, self-controlled clinical trial
Indication:	Bilateral primary inguinal hernias
Type of examination, study design	Prospective, self-controlled, two-arm comparative study of two CE-certified medical devices in the intended indication with regard to efficacy.
Number of patients:	496 patients with bilateral inguinal hernia→ 992 hernias
Primary study objective:	The aim is to show that the use of biological mesh material for laparoscopic hernia treatment results in significantly less postoperative pain than the use of synthetic mesh material, without an increased recurrence rate.
Target values:	Primary targets:<br>• Pain after 6 months measured with a visual analogue scale (VAS 0-10)<br>• Recurrence rate after 2 years<br>Secondary targets:<br>• Frequency and intensity of pain at other time points (one week and one year after surgery)<br>• Recurrence rate after one year<br>• Local infection of the surgical site<br>• Mesh dislocations<br>• Hematoma<br>• Seroma<br>• Patient satisfaction (overall result, foreign body sensation, paresthesia, one week and one year after surgery)

Evaluation criteria:	Effectiveness:<br>• The expected advantage of the biological mesh is 0.5 points on the 0-10 VAS.<br>• An assumed recurrence rate after 2 years of 5% for the synthetic mesh material contrasts with a recurrence rate of ≤8% for the biological mesh material.<br>Security:<br>• Number and type of complications such as re-interventions or replacement of the mesh
Diagnosis and main inclusion criteria:	Main inclusion criterion:<br>• Patients with bilateral primary inguinal hernias<br>• Patient is suitable for laparoscopic surgery<br>Main exclusion criteria:<br>• Recurrent hernia<br>• Incarcerated hernia<br>• Presence of an acute systemic infection
Medical product: Application type:	Not two specific products but two types of material are compared. Each study center must use commercially available mesh material. However, the following requirements apply:<br>• CE-certified meshes<br>• The synthetic mesh materials should be made of polypropylene, polyester or PVDF, have large pores and be lightweight (< 100 g/m^2).<br>• The biological mesh material should consist of a perforated, non-crosslinked, acellular and collagenous matrix<br>• The meshes should have a minimum size of 10x15 cm<br>• The meshes can be fixed in place
Comparative therapy, dosage and method of application:	Both mesh materials are used for each patient and compared directly with each other (self-controlled design). Other procedures/materials are not usual for this indication. The usual laparoscopic implantation in hernia repair is performed in accordance with IEHS guidelines.
Duration of therapy:	The duration of treatment corresponds to the clinical routine and takes about 1 hour for the procedure itself. Both hernias are treated during one procedure.<br>Follow-up examinations are carried out up to 2 years after the procedure

2. Statistical analysis

Hernias, not patients, are randomised. In each patient, one hernia is treated with synthetic mesh and the hernia on the other side is treated with biological mesh. In this "self-controlled design", each patient is his or her own control.

The decision as to whether the left or right hernia is operated on with the biological mesh is randomised. The study unit is therefore not the patient, but the individual hernia. The test for comparability of the two "study groups" is therefore limited to the size of the hernia and the pre-operative pain, which is recorded separately for each side.

2.1 Study population

All patients who have been informed and whose written consent has been obtained will be included in the study. Furthermore, both hernias must have been surgically treated.

If patients have only undergone unilateral surgery (or no surgery at all), these patients are listed with the number and reason, but excluded from further analyses.

All endpoints are analysed according to the intention-to-treat principle. The unit of analysis is the hernia, not the patient. As only patients operated on both sides are analysed, the number of cases is identical for both mesh materials.

Protocol breaches with regard to therapy allocation can be
- both sides were treated with the same mesh material (biological or synthetic)
- the treatment was carried out in exactly the opposite way to that intended in the randomisation.

Such protocol breaches are enumerated (n, %). If there is a deliberate deviation from the randomisation specification, a reason must be given. For the ITT evaluation, the assignment is made according to the randomisation, regardless of the mesh actually used.

If more than 2% of patients are affected by such protocol breaches, a Per Protocol (PP) and an As Treated (AT) analysis is also performed for the primary and secondary outcomes.

Patient inclusion over time is shown graphically; the date of the operation is decisive.

2.2 Visits

During the course of the study, visits were made at seven defined points in time:

Visit 1: Screening

Visit 2: Surgery

Visit 3: Discharge

Visit 4: Follow-up 1 week after surgery

Visit 5: Follow-up 6 months after surgery

Visit 6: Follow-up 12 months after surgery

Visit 7: Follow-up 24 months after surgery

The presence of the individual visits is shown in the appendix for each individual patient. The follow-up rates for visits 4, 5, 6 and 7 are summarised. For the visits performed, the period since the operation is calculated (in months, except visit 4 in days): Mean, SD, median, minimum, maximum.

2.3 Describing the patients

The following data are presented to describe the included patients: (categorical characteristics with n/%; metric characteristics with mean, median and standard deviation (SD))

- Age (years)
- Gender
- Height (cm)
- Weight (kg)
- BMI
- ASA
- Previous operations
- Concomitant diseases
- Time between randomisation and surgery
- Length of hospitalisation (in days from surgery)
- General complications:
  - Systemic infection
  - Ileus
  - Revision surgery

Intraoperative data:

- Simultaneous interventions
- Intraoperative complications (yes/no and type)
- Antibiotic prophylaxis
- Thrombosis prophylaxis
- Drainage
- If yes, duration of drainage (days)
- Adhesiolysis
- OP duration (min.)
- Complications (yes/no)

2.4 Description of hernias

In order to compare the two hernias (left/right), groups are formed with regard to the randomised therapy (biological versus synthetic mesh) and the following characteristics are presented descriptively for each group:

- Size of the hernia
- right / left
- Grading (1/2/3)
- Pain at rest (VAS)
- Pain under stress (VAS)

Intra-operative findings:

- Implanted mesh material
- Use of adhesive

Findings on discharge (visit 3):

- Complication (yes/no)
- If yes: - Haematoma formation (with/without intervention)
  - Seroma formation (with/without intervention)
  - Mesh dislocation / early recurrence

The meshes used (synthetic/organic) are listed by manufacturer.

2.5 Follow-up; primary endpoints

Pain

At the time of discharge (visit 3) and at the 4 follow-up examinations (after 1 week and after 6, 12, 24 months; visits 4-7), pain at rest and under stress was documented using a visual analogue scale (VAS; 0-10). Pain under stress was used to test the primary endpoint.

If there are no follow-up examinations, the LOCF principle (Last Observation Carried Forward) is applied and the value of the previous examination is used. This principle is also applied backwards, i.e. if a 12-month result is available but earlier results are not, the 12-month data is also added "backwards".

Patients who do not have a pain score at any of the examination times (visits 3-7) are excluded from this analysis (number is reported). Missing values at visit 3 (discharge) and after 1 week (visit 4) can replace each other in the event of missing values.

Pain is always recorded separately for the left and right side. If only one pain value is available (i.e. only for RIGHT or LEFT), the missing value is replaced by the value for the other side. If both pain values are missing, they are replaced according to LOCF as described above.

The primary outcome measure is pain under stress after 6 months (visit 5). This is tested confirmatory with the Wilcoxon signed-rank test for paired samples in a comparison of the two mesh materials (α = 0.025).

Pain at rest and pain under stress at the other time points are also compared as secondary outcome measures. The descriptors are mean, median, range and SD.

Recurrences

At least one follow-up examination is required for the evaluation of the endpoint "recurrence" (visit 4-7). Patients without a follow-up are listed and excluded from the following analysis of recurrences.

The occurrence of a recurrence is recorded separately for each side and requires diagnostic confirmation.

For the primary comparison of recurrence rates after implantation of a biological or plastic mesh, the findings at 2 years after the operation are evaluated. If a (confirmed) recurrence is

present at this time, recurrence is set to "yes". This is also the case if a recurrence was detected at an earlier point in time. If there is no 2-year follow-up, the most recent result is used instead; this corresponds to the LOCF (*last observation carried forward*) procedure. So if a patient has their last examination result after one year and the 2-year follow-up is missing, the result after one year is used.

The recurrence rate after two years is determined for both mesh materials and presented with 95 % confidence intervals. The difference between the two recurrence rates is then determined from the two rates (biological mesh - synthetic mesh). A positive difference therefore means a higher recurrence rate for the biological mesh, a negative value means a lower recurrence rate. A 95 % confidence interval is then also calculated for this difference.

If the value of 3.0 % lies within this confidence interval for the difference, it cannot be ruled out with certainty that biological meshes have a higher recurrence rate; in this case, equivalence (or better, non-inferiority) would not be given. However, if the value of 3.0 % lies outside the confidence interval, non-inferiority can be assumed with a high degree of certainty (in this case 2.5 %).

Descriptively, the recurrences are shown cumulatively with the help of a Kaplan-Meier curve.

2.6 Safety

The following complications are listed separately for both sides (mesh materials):

- Infection
- Hematoma/bleeding
- Seroma formation
- Mesh dislocation
- Revision operation

The frequency of occurrence is compared using the McNemar test for event rates in paired data.

The following complications are reported at patient level, as they cannot be reliably assigned to one side or one material:

- Intraoperative complications (hemorrhage; injury to bowel or bladder; conversion)
- Systemic infection
- Ileus

Perioperative thrombosis prophylaxis and the administration of antibiotics are presented at patient level. This also applies to the insertion of a drain and any necessary adhesiolysis.

The cumulative number of hospitalisations is also shown at patient level.

2.7 Secondary endpoints

The following endpoints are compared as secondary endpoints between the two mesh materials:

2.7.1 Recurrences after one year

The evaluation is carried out analogue to the primary endpoint

2.7.2 Pain

Pain at rest and under stress at all visits (3 - 7)
The evaluation takes place parallel to the primary endpoint

2.7.3 Satisfaction

Satisfaction with the treatment is surveyed separately for each side on discharge and in the follow-up. This includes
- foreign body sensation (yes/no)
- paresthesia (yes/no)

In addition, the patient is asked which side they are more satisfied with (the patient can also answer "no difference"). As the patient is blinded to the type of material, this represents an objective comparison of the two materials.
The frequencies for the answers "Bio. Mesh better", "no difference" and "Synth. Mesh better" are reported.
For statistical testing, a point score
is determined (+1 / 0 / -1) and the probability for the calculated number
is determined according to a binomial distribution ($B_{n,p}$), assuming that p=0.5.

2.8 Database

The study data is summarised in an SPSS file. The currently available version of SPSS (IBM Inc., Armonk, NY, USA) is used for the analysis.
