# Supplementary material for: Biological vs Synthetic Mesh in Laparoendoscopic Inguinal Hernia Repair: The BIOLAP Randomized Clinical Trial
Source: JAMA Surg. 2025 Oct 8;160(12):1309–16. doi: 10.1001/jamasurg.2025.4071 (PMC12509081; doi:10.1001/jamasurg.2025.4071)
Supplement: Supplement 3. — eAppendix. List of Study Investigators and Sites eTable 1. Hernia Subtypes eTable 2. Postoperative Complications eTable 3. Biological and Synthetic Meshes Used in the BIOLAP Trial eTable 4. Recurrence and Patient Age eTable 5. Seroma and Hematoma eTable 6. Postoperative Paresthesia eTable 7. Postoperative Foreign Body Sensation eTable 8. Postoperative Patient Satisfaction [file jamasurg-e254071-s003.pdf]

Supplemental Online Content

Seefeldt CS, Knievel J, Deeb M, et al. Biological vs synthetic mesh in laparoendoscopic inguinal hernia repair: the BIOLAP randomized clinical trial. *JAMA Surg*. Published online October 8, 2025. doi:10.1001/jamasurg.2025.4071

eAppendix. List of Study Investigators and Sites

eTable 1. Hernia Subtypes

eTable 2. Postoperative Complications

eTable 3. Biological and Synthetic Meshes Used in the BIOLAP Trial

eTable 4. Recurrence and Patient Age

eTable 5. Seroma and Hematoma

eTable 6. Postoperative Paresthesia

eTable 7. Postoperative Foreign Body Sensation

eTable 8. Postoperative Patient Satisfaction

This supplemental material has been provided by the authors to give readers additional information about their work.

© 2025 Seefeldt CS et al. *JAMA Surgery*.

eAppendix. List of Study Investigators and Sites

Krankenhaus Köln-Merheim, Kliniken der Stadt Köln: Christian Fischer-Sille, Mathias Formesyn, Kathrin Heidemeier, Markus M. Heiss, Jonas Lange, Philipp Meibert, Jürgen Meyer-Zillekens, Claudia Simone Seefeldt, Panagiotis Thomaidis, Friederike Weber, Niklas Weltermann

Asklepios Westklinikum Hamburg, St. Elisabeth Krankenhaus Thuine: Thomas Carus, Peter Pick

Hernienzentrum, PAN-Klinik Köln: Alexander Digón Sönthgerath, Bernd Stechemesser

St. Elisabeth-Krankenhaus Dorsten: Judith Beckfeld, Ruslan Chirikov, Wilhelm Gross-Weege, Ethem Topsakal

GFO Kliniken Rhein Berg Bergisch Gladbach: Peter C. Ambe, Ulrich Böhm, Ralf Essen, Maria-Eleni Novsinanov, Wolfgang Spangenberger

St. Marien-Krankenhaus Ahaus: Frederik Amm, Julien Holtermann, Mirela Karamehmedovic, Moritz Meyer, Erik Paust, Makaju Shrestha, Ciprian Zaharia

Helios Klinik Attendorn: Mathias Bebobru, Klaus Friedhoff, Heidi Hoffmann, Ahmad Farid Nasri, Saliman Ziai

Johanniter-Krankenhaus Bonn: Nicola Cerasani, Luzi Westphal

Josephs-Hospital Warendorf: Ahmed Al-Badani, Lothar Biermann, Leonid Bograd, Horst Dübner, Wolfgang Gänsler, Sebastian Hofmeister, Andrejus Kriukovas, Johann Malath, Barbara Schulze Eilfing, Christoph Seiler

Klinikum Leverkusen: Dirk Antoine, Nico Schäfer

KMG Klinikum Luckenwalde: Jan Dornbusch, Oliver Eckermann, Ulrich Fleck, Frank Schischke

St. Barbara-Klinik Hamm-Heessen: Christoph Bonk, Dietmar Picke

GRN Klinik Weinheim: Oliver Hauer, Frauke Hildebrandt, Alexandra Mandry, Angela Ruppert-Notz, Thomas Simon, Torsten Wilhelm

Evangelisches Diakonissenkrankenhaus Leipzig: Charlotte Brat, Janina Dengler, Richard Gnatzy, Niels-Torsten Hoedt, Andreas Legler, Shueb Mussa, Steffen Otte, Jan Zabel

Eduardus-Krankenhaus Köln-Deutz: Thomas Kröger, Alexander Krökel, Jörg Weber, Andreas Wölk

Lukaskrankenhaus Neuss: Andreas Bär, Anna Dobek-Witkowska, Bernhard Lammers, Sebastian Wais

University Hospital RWTH Aachen: Roman Eickhoff, Christian Klink, Andreas Lambertz, Daniel Antonio Morales Santana, Mark Schneider

Evangelisches Klinikum Köln Weyertal: Michael Arbogast, Randi Bieling, Regina Birkner, Bruno Diemer, Carina Heider, Joshy Madukkakuzhy, Desiree Müller, Jakob Otten, Claudia Rudroff, Christoph Ulrici, Alberto Vega Hernandez, Mark Weber

St. Bernhard-Hospital Kamp-Lintfort: Mark Banysch, Sebastian Bochlogyros, Carolyn Borman, Claudio Brauner, Bianca Claassens, Radoslav Doneber, Oleg Gurenko, Ilham Herrmann, Hannes Irmer, Gernot Kaiser, Attaher Madou, Katja Marx, Genadi Mero, Stanislav Pasamonik, Hülya Pusta

Ammerland Klinik Westerstede: Muneer Deeb, Andreas Rackwitz, Lars Zens

Dreifaltigkeits-Krankenhaus Wesseling: Julia Brandt, Christoph Jacobi, Rainer Langeder

© 2025 Seefeldt CS et al. *JAMA Surgery*.

eTable 1. Hernia Subtypes

All subtypes of the European Hernia Society's primary inguinal hernia classification were included in the trial, n=982

	L0	L1	L2	L3
**M0**		159	275	70
**M1**	53	43	39	6
**M2**	136	57	46	7
**M3**	55	15	17	4

eTable 2. Postoperative Complications

Additional postoperative complications which were defined as 'other' in the manuscript, table 1

Other postoperative complications until discharge	n (%)
Swelling of the spermatic cord	5 (1)
Pain	2 (0.4)
Urinary retention	2 (0.4)
Urosepsis in urolithiasis	1 (0.2)
Rash shoulder/neck	1 (0.2)
Hemorrhage skin naevus	1 (0.2)
Intestinal colic with intestinal atony	1 (0.2)
Fever	1 (0.2)
Extensive hematoma in the middle	1 (0.2)
Leukocytosis	1 (0.2)
Pain event left lower abdomen with collapse	1 (0.2)
Intraoperative opening of the bladder	1 (0.2)

© 2025 Seefeldt CS et al. *JAMA Surgery*.

eTable 3. Biological and Synthetic Meshes Used in the BIOLAP Trial

The trial sites could use all Conformité Européenne (CE)-certified, commercially available meshes that are at least 10 x 15 cm in size. The biological mesh should be a perforated, non-cross-linked, acellular, collagenous matrix. The synthetic mesh should be large-pored, lightweight and made of polypropylene, polyester, or polyvinylidene fluoride.

Biological Meshes	n (%)
Biodesign® Inguinal Hernia Graft, Cook Biotech Incorporated	248 (50.5)
SurgiMend®, Integra LifeSciences	230 (46.8)
Tutomesh®, Tutogen Medical GmbH	13 (2.7)

Synthetic Meshes	n (%)
3DMax™ Light Mesh, Bard Davol Inc.	123 (25.1)
Ultrapro Advanced™/Ultrapro®, Ethicon	122 (24.9)
DynaMesh®-Endolap/-Endolap 3D, DynaMesh	93 (18.9)
ProGrip™, Medtronic GmbH	48 (9.8)
Optilene® Mesh, B.Braun	31 (6.3)
Prolene® Soft, Ethicon	28 (5.7)
Parietene™, Medtronic GmbH	27 (5.5)
TiO_2Mesh™, BioCer GmbH	11 (2.2)
Bard® SoftMesh, Bard Davol Inc.	8 (1.6)

eTable 4. Recurrence and Patient Age

Mean and median age of patients with and without recurrence

Mesh	Recurrence	Number	Age Mean/median (SD)
Biological	No recurrence	438	57.6 / 57 (14.1)
	Recurrence	53	61.6 / 60 (10.8)
Synthetic	No recurrence	479	58.0 / 58 (14.0)
	Recurrene	12	60.1 / 61 (15.7)

© 2025 Seefeldt CS et al. *JAMA Surgery*.

eTable 5. Seroma and Hematoma

Occurrence of seromas and hematomas, diagnosed at least once during the follow-up visits. Shown as number, ratio (%) and CI_{95}.

N	Bio. mesh	Synth. mesh	bilateral	p-value
**Seroma**				
491	n=164<br>33.4%<br>28.5 – 38.9	n=106<br>21.6%<br>17.7 – 25.2	n=70	<.001
**Hematoma**				
491	n=72<br>14.7%<br>11.5 – 18.5	n=59<br>12.0%<br>9.2 – 15.5	n=29	.160

eTable 6. Postoperative Paresthesia

Patients were asked about the occurrence of paresthesia at each post-operative visit. Hernias with a recurrence were not followed up. Data is also missing because patients did not attend the visits or did not provide any information.

Paresthesia		Biological Mesh n (%)	Synthetic Mesh n (%)	p-value
Visit 3 discharge	No	482 (98.4)	483 (98.6)	.795
	Yes	8 (1.6)	7 (1.4)	
	Total	490 (100)	490 (100)	
Visit 4 1 week	No	447 (94.7)	459 (93.5)	.071
	Yes	25 (5.3)	14 (2.9)	
	Total	472 (100)	473 (100)	
Visit 5 6 months	No	425 (95.1)	423 (96.9)	.176
	Yes	22 (4.9)	14 (3.1)	
	Total	447 (100)	447 (100)	
Visit 6 12 months	No	416 (99.0)	429 (98.6)	.562
	Yes	4 (1.0)	6 (1.4)	
	Total	420 (100)	435 (100)	
Visit 7 24 months	No	380 (98.4)	406 (98.5)	.909
	Yes	6 (1.6)	6 (1.5)	
	Total	386 (100)	412 (100)	

© 2025 Seefeldt CS et al. *JAMA Surgery*.

eTable 7. Postoperative Foreign Body Sensation

Patients were asked about foreign body sensation at each post-operative visit. Hernias with a recurrence were not followed up. Data is also missing because patients did not attend the visits or did not provide any information.

foreign body sensation		Biological Mesh n (%)	Synthetic Mesh n (%)	p-value
Visit 3 discharge	No	470 (95.9)	478 (97.6)	.150
	Yes	20 (4.1)	12 (2.4)	
	Total	490 (100)	490 (100)	
Visit 4 1 week	No	424 (89.6)	443 (93.7)	.025
	Yes	49 (10.4)	30 (6.3)	
	Total	473 (100)	473 (100)	
Visit 5 6 months	No	421 (94.2)	424 (95.1)	.558
	Yes	26 (5.8)	22 (4.9)	
	Total	447 (100)	446 (100)	
Visit 6 12 months	No	404 (96.2)	420 (96.6)	.778
	Yes	16 (3.8)	15 (3.4)	
	Total	420 (100)	435 (100)	
Visit 7 24 months	No	383 (99.2)	402 (97.6)	.066
	Yes	3 (0.8)	10 (2.4)	
	Total	386 (100)	412 (100)	

© 2025 Seefeldt CS et al. *JAMA Surgery*.

eTable 8. Postoperative Patient Satisfaction

Patients were asked at each post-operative visit whether they were more satisfied with one side. The patients did not know which mesh material was implanted on which side. P-value was calculated using the McNemar test.

Patient satisfaction	Biological Mesh n (%)	Synthetic Mesh n (%)	Equal n (%)	Total n (%)	p-value
Visit 3 discharge	61 (12.5)	74 (15.1)	354 (72.4)	489 (100)	.302
Visit 4 1 week	90 (19)	150 (31.7)	233 (49.3)	473 (100)	<.001
Visit 5 6 months	79 (17.6)	90 (20)	281 (62.4)	450 (100)	.442
Visit 6 12 months	64 (14.6)	84 (19.2)	290 (66.2)	438 (100)	.118
Visit 7 24 months	56 (13.5)	69 (16.7)	289 (69.8)	414 (100)	.283

© 2025 Seefeldt CS et al. *JAMA Surgery*.
