# Supplementary material for: Biological vs Synthetic Mesh in Laparoendoscopic Inguinal Hernia Repair: The BIOLAP Randomized Clinical Trial
Source: JAMA Surg. 2025 Oct 8;160(12):1309–16. doi: 10.1001/jamasurg.2025.4071 (PMC12509081; doi:10.1001/jamasurg.2025.4071)
Supplement: Supplement 4. — Data Sharing Statement [file jamasurg-e254071-s004.pdf]

Data Sharing Statement

Seefeldt. Biological vs Synthetic Mesh in Laparoendoscopic Inguinal Hernia Repair. *JAMA Surg*. Published October 08, 2025. doi:10.1001/jamasurg.2025.4071

Data
Additional Information: German Clinical Trials Register DRKS00010178
https://www.drks.de/search/de/trial/DRKS00010178/details
Data available: Yes
Data types: Deidentified participant data
How to access data: To access data, a request should be submitted to the corresponding author with a scientific proposal including objectives. Data will only be shared after a data sharing agreement is fully executed.
When available: With publication

Supporting Documents
Document types: Statistical/analytic code
How to access documents: To access data, a request should be submitted to the corresponding author with a scientific proposal including objectives. Data will only be shared after a data sharing agreement is fully executed.
When available: With publication

Additional Information
Who can access the data: researchers whose proposed use of the data has been approved
Types of analyses: for a specified purpose
Mechanisms of data availability: after approval of a proposal, or with a signed data access agreement
